# Suppression of Interferon Lambda Signaling by SOCS-1 Results in Their Excessive Production during Influenza Virus Infection

**DOI:** 10.1371/journal.ppat.1003845

**Published:** 2014-01-02

**Authors:** Haitao Wei, Song Wang, Qinghuang Chen, Yuhai Chen, Xiaojuan Chi, Lianfeng Zhang, Shile Huang, George F. Gao, Ji-Long Chen

**Affiliations:** 1 CAS Key Laboratory of Pathogenic Microbiology and Immunology, Institute of Microbiology, Chinese Academy of Sciences, Beijing, China; 2 College of Animal Science, Fujian Agriculture and Forestry University, Fuzhou, China; 3 Key Laboratory of Human Disease Comparative Medicine, Ministry of Health, Institute of Laboratory Animal Science, Chinese Academy of Medical Sciences & Comparative Medical Center, Peking Union Medical College, Beijing, China; 4 Department of Biochemistry and Molecular Biology, Louisiana State University Health Sciences Center, Shreveport, Louisiana, United States of America; Harvard Medical School, United States of America

## Abstract

Innate cytokine response provides the first line of defense against influenza virus infection. However, excessive production of cytokines appears to be critical in the pathogenesis of influenza virus. Interferon lambdas (IFN-λ) have been shown to be overproduced during influenza virus infection, but the precise pathogenic processes of IFN-λ production have yet to be characterized. In this report, we observed that influenza virus induced robust expression of IFN-λ in alveolar epithelial cells (A549) mainly through a RIG-I-dependent pathway, but IFN-λ-induced phosphorylation of the signal transducer and activator of transcription protein 1 (STAT1) was dramatically inhibited in the infected cells. Remarkably, influenza virus infection induced robust expression of suppressor of cytokine signaling-1 (SOCS-1), leading to inhibition of STAT1 activation. Interestingly, the virus-induced SOCS-1 expression was cytokine-independent at early stage of infection both *in vitro* and *in vivo*. Using transgenic mouse model and distinct approaches altering the expression of SOCS-1 or activation of STAT signaling, we demonstrated that disruption of the SOCS-1 expression or expression of constitutively active STAT1 significantly reduced the production of IFN-λ during influenza virus infection. Furthermore, we revealed that disruption of IFN-λ signaling pathway by increased SOCS-1 protein resulted in the activation of NF-κB and thereby enhanced the IFN-λ expression. Together, these data imply that suppression of IFN-λ signaling by virus-induced SOCS-1 causes an adaptive increase in IFN-λ expression by host to protect cells against the viral infection, as a consequence, leading to excessive production of IFN-λ with impaired antiviral response.

## Author Summary

Influenza virus infection triggers innate immune responses. However, aberrant host immune responses such as excessive production of cytokines contribute to the pathogenesis of influenza virus. Type III interferons (IFN-λ) constitute the major innate immune response to influenza virus infection, but the precise pathogenic processes of IFN-λ production and mechanistic underpinnings are not well understood. In this study, we report that influenza virus induces robust IFN-λ expression mainly through a RIG-I-dependent pathway, but signaling activated by IFN-λ was dramatically inhibited by virus-induced SOCS-1. Importantly, we found that disruption of the SOCS-1 expression or forced activation of STAT1 significantly reduced the expression of IFN-λ *in vitro* and *in vivo*, suggesting that suppression of IFN-λ signaling by SOCS-1 results in their excessive production during influenza virus infection. Furthermore, our experiments revealed that disruption of IFN-λ signaling pathway resulted in the activation of NF-κB that governs the IFN-λ expression. Together these findings, we propose that impaired antiviral response of IFN-λ due to the inhibitory effect of SOCS-1 causes an adaptive increase in IFN-λ expression by host to protect cells against the viral infection. This is a novel mechanism that may be critical in the pathogenesis of the influenza virus strains that induce hypercytokinemia.

## Introduction

Influenza A virus (IAV), a highly infectious respiratory pathogen, causes worldwide annual epidemics and occasional pandemics. Therefore, IAV has continued to be a top global public health threat. The host cytokine immune response provides the first line of defense against IAV infection. A variety of cell types in the host, including activated alveolar macrophages (AM), lymphocytes, dendritic cells (DC), lung alveolar epithelial cells and endothelial cells within lung tissue, produce cytokines and chemokines following IAV infection, thus playing key roles in innate and adaptive immunity [1]–[3]. However, an aberrant innate response, with early recruitment of inflammatory leukocytes to the lung, was believed to contribute to the morbidity of the 1918 influenza virus infection [4]. Studies have also shown that highly virulent influenza virus infection induces excessive cytokine production (cytokine storm) and robust recruitment of leukocytes which are hypothesized to be major contributors to severe disease in humans from influenza virus infection [5]. These data reveal that dysregulation of cytokine signaling of the host during influenza virus infection caused by inappropriate activation of the innate immune response triggers massive pulmonary injury and immune-mediated organ dysfunction. However, the mechanisms underlying the increased induction of innate immune cytokines during influenza virus infection have to date been largely unclear.

Innate immune responses triggered by the intracellular detection of viral infections include the production of interferons (IFNs) that are classified within the class II cytokine family based on the similarity of their receptors. IFNs consist of three types of cytokines: type I IFNs include IFN-α and IFN-β; type II IFN is IFN-γ and type III IFNs consist of three members in humans, IFN lambda1 (IFN-λ1), IFN-λ2, and IFN-λ3 which are also named IL-29, IL-28A, and IL-28B, respectively, whereas mice only express IFN-λ2 and IFN-λ3 [6]. Virus-infected cells secrete a complex mixture of IFNs that represent a major element of the innate immune response against diverse viral infections [7]. In 2003, IFN-λs were first discovered as novel antiviral cytokines by two independent groups [8], [9]. It is now recognized that IFN-λs are virus-induced cytokines with type I IFN-like biological functions, including antiviral activity, but have evolved independently of type I IFNs [10]. Although both type I IFNs and IFN-λ are expressed by a host in response to viral infections, IFN-λs, not type I IFNs, are the predominant IFNs induced by respiratory viruses in nasal epithelial cells and mainly contribute to the first line defense against respiratory virus infection [11]. Type I IFNs were first recognized for their ability to interfere with IAV replication, but IFN-λs have recently been shown to be present at much higher levels than type I IFNs in the lungs of infected mice and play an important role in host defense against IAV infection [2]. However, currently there is limited information available about the biology of IFN-λ. In particular, the mechanisms that regulate the robust expression of IFN-λ during IAV infection are not fully understood.

IFN-λs share a common cellular receptor consisting of the cytokine receptor family class II members IL-28R1 and IL-10R2. The short chain IL-10R2 is ubiquitously expressed and is a receptor component of other type II-related cytokines, whereas the long chain IL-28R1 is unique to IFN-λ and is preferentially expressed on epithelial cells [12]. IFN-λs are induced by most, if not all, classes of viruses as well as some bacterial products [10]. Once secreted, IFN-λs act in an autocrine or paracrine manner by binding the cell-surface receptors. The receptor binding results in a conformational change in the receptor and activation of the receptor-associated Janus tyrosine kinases (JAKs). Activated JAK1 and Tyk2 transphosphorylate the receptor chains that assist in the recruitment of STAT proteins. STAT proteins are then phosphorylated, dimerized, and translocated to the nucleus to initiate transcription of the IFN-stimulated genes (ISGs) that mediate the biological effects of IFN-λ. Therefore, IFN-λ-mediated activation of JAK/STAT signaling is required for efficiently triggering the synthesis of antiviral factors.

An important mechanism for negative regulation of the JAK/STAT signaling pathway is mediated through members of the SOCS family. Of the eight family members, SOCS-1 has been most extensively studied and is the most potent inhibitor of cytokine-induced signaling [13]. SOCS-1 can directly interact with JAKs by its kinase inhibitory region (KIR), which inhibits JAKs activity. In addition, SOCS-1 can target JAKs to proteasomal degradation through interaction of SOCS box with the Elongin BC complex, which becomes part of an E3 ubiquitin ligase [14]–[16]. When overexpressed in cells, SOCS-1 can inhibit STAT activation induced by multiple cytokine stimulations. Interestingly, several recent studies have revealed that influenza virus has developed mechanisms to subvert host antiviral defense mediated by type I and type II IFNs through inhibition of the JAK/STAT signaling by upregulated SOCS-1 and SOCS-3 proteins [17]–[20]. Consistent with these observations, it has been shown that IFN-λ-induced mRNA expression of the antiviral proteins 2′,5′-OAS and Mx1 was abolished by overexpression of SOCS-1 [21]. However, the relationship between suppression of cytokine signaling by SOCS-1 and overproduction of IFN-λ during influenza virus infection remains to be determined.

In this study, we examined the effects of influenza virus-provoked negative regulation of cytokine signaling on the IFN-λ production by altering expression of SOCS-1 and activation of STAT signaling. We found that disrupting SOCS-1 expression or constitutive activation of STAT1 significantly inhibits production of IFN-λ *in vitro* and *in vivo*. The results reveal that suppression of innate immune cytokine signaling by virus-induced SOCS-1 contributes to formation of IFN-λ storm during influenza virus infection.

## Results

### Intracellular detection of IAV infection induces robust expression of IFN-λ in alveolar epithelial cells mainly through a RIG-I-dependent pathway

**Figure 1:**
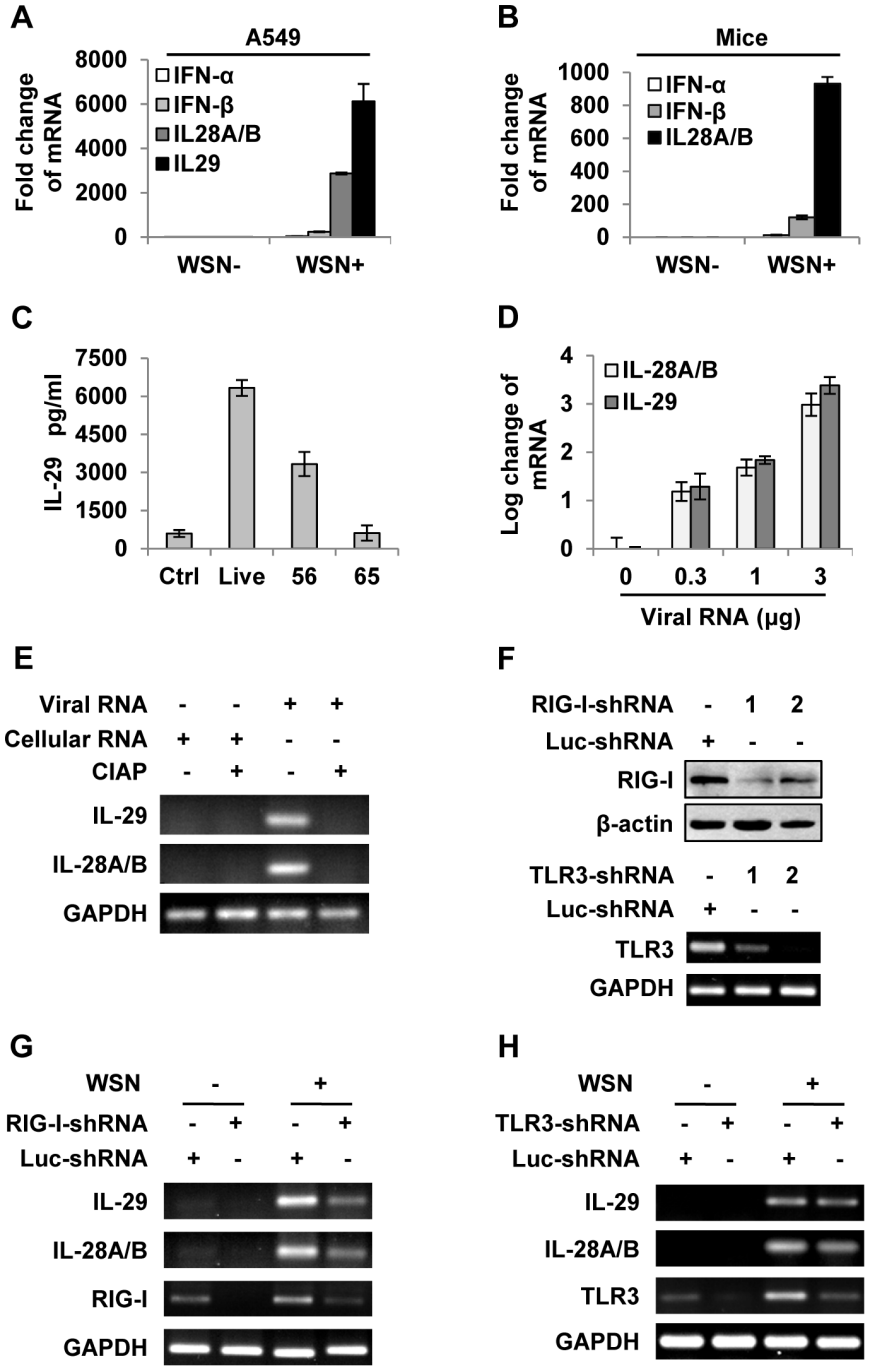
IAV infection induces robust expression of IFN-λ in alveolar epithelial cells mainly through a RIG-I-dependent pathway. (**A**) A549 cells infected with or without WSN virus (MOI = 1) for 15 h, mRNA levels of IFN-α, β and λ were examined by real-time PCR. (**B**) BALB/c mice were infected intranasally with or without WSN virus (1×10^5^ PFU). On day 3 p.i., lungs were lysed, and the mRNA levels of IFN-α, β and λ were examined by real-time PCR. (**C**) A549 cells were uninfected (Ctrl) or infected with WSN that was untreated (Live) or treated at 56°C or 65°C. IL-29 levels in supernatants from A549 cells at 15 h p.i. were measured by ELISA. (**D**) Different amounts of total RNA (“Viral RNA”) from A549 cells infected with the IAV were transfected into native A549 cells using Lipofectamine 2000 (L2000). Expression of IL-28A/B and IL-29 in transfected A549 cells was examined by real-time PCR at 4 h p.i. (**E**) “Viral RNA” and “Cellular RNA” (total RNA from uninfected control A549 cells) treated with or without calf intestine alkaline phosphatase (CIAP) were transfected into native A549 cells. RT-PCR was performed to examine the expression of IL-28A/B and IL-29. (**F**) shRNA based-knockdown of RIG-I and TLR3 were analyzed by Western blotting or RT-PCR to determine the interference efficiency. (**G–H**) A549 cells expressing shRNAs targeting RIG-I (G), TLR3 (H) or luciferase (Luc) were infected with or without WSN, and then the expression of IL-28A/B and IL-29 was examined by RT-PCR. Results are representative of three independent experiments.

To investigate the mechanisms by which the host cells interact with influenza A virus (IAV), we have recently used cDNA microarray to profile the cellular transcriptional response to A/WSN/33 influenza virus (H1N1) infection in A549 human alveolar epithelial cells [22]. Surprisingly, we found that IL-28A, IL-28B and IL-29, three recently discovered IFN-λ family members, were most significantly up-regulated (Figure S1A). This finding was confirmed by independent experiments measuring the mRNA levels by quantitative real time PCR of IAV infected A549 cells and mouse lungs (Figure 1A and B) and RT-PCR (Figure S1B), and evaluating the IL-29 protein level by ELISA (Figure S1C). Treatment of IAV at 56°C for 30 minutes, which prevents viral replication without affecting viral entry into host cells [23], significantly reduced the virus-induced production of IFN-λs (Figure 1C). To further determine whether production of IFN-λ was affected by viral entry into host cells, IAV was inactivated at 65°C for 30 minutes, which denatures hemagglutinin (HA) and prevents host cell attachment [24]. We found that expression of IFN-λ induced by 65°C-inactivated virus recapitulated that of the non-infected control (Figure 1C). These experiments demonstrated that robust expression of IFN-λs was the response to live virus entry into host cells and viral replication.

To further determine the inducer of IFN-λs, A549 cells were transfected with either different amounts of total RNA isolated from IAV infected cells (Figure 1D and Figure S1D) or genomic RNA directly isolated from the viruses (Figure S1E). The results revealed that both viral genome RNA and viral RNA generated during IAV infection contributed to IFN-λ production. Unlike cellular RNA, influenza viral RNA contains a 5′-triphosphate group which is thought to be the critical trigger for production of type I IFNs through RIG-I-dependent pathway [25]–[27]. Using calf intestine alkaline phosphatase (CIAP) to remove the 5′-triphosphate terminus of viral RNA, we tested whether it was involved in IFN-λ induction. Interestingly, treatment with CIAP greatly inhibited expression of IFN-λs (Figure 1E and Figure S1F). To determine whether IAV-induced expression of IFN-λ was completely dependent on RIG-I, A549 cell lines stably expressing shRNAs targeting either RIG-I, TLR3 or MDA5 were generated (Figure 1F and Figure S1G). We observed that silencing RIG-I resulted in a marked decrease in the production of IFN-λ and silencing TLR3 slightly decreased the IFN-λ levels, whereas disruption of MDA5 expression had no overt effects on the IFN-λ production (Figure 1G–H and Figure S1G–H). These data suggest that IFN-λ induced by the IAV RNA was mainly through a pathway involving RIG-I.

### IAV inhibits IFN-λ-stimulated STAT1 phosphorylation in host

**Figure 2:**
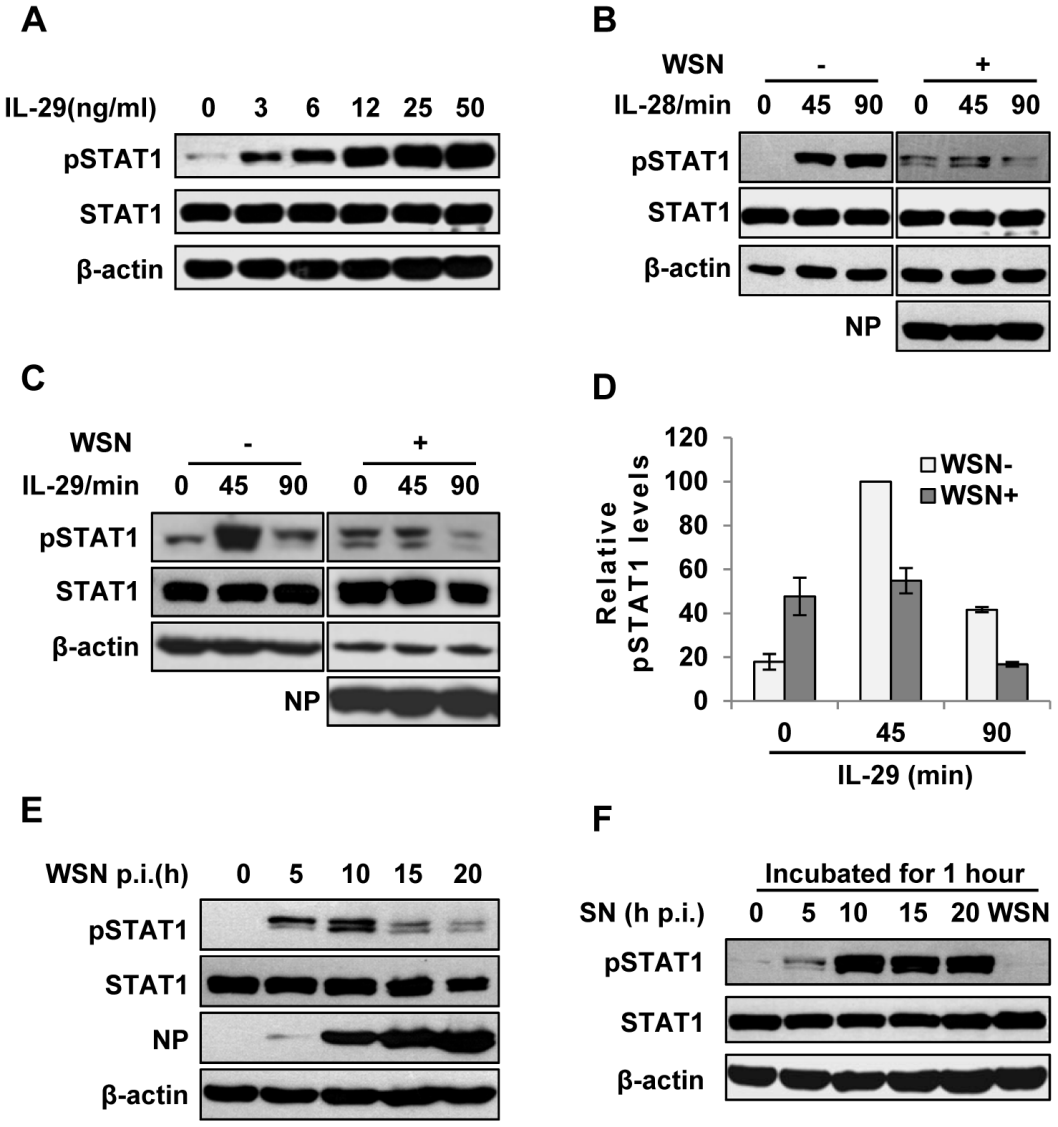
IAV inhibits IL-29-induced STAT1 phosphorylation in A549 cells. (**A**) A549 cells were treated with IL-29 at final concentration of 3, 6, 12, 25, and 50 ng/ml for 45 min, followed by immunoblotting with indicated antibodies. (**B, C**) A549 cells infected with WSN (MOI = 1) for 15 h (WSN+) or non-infected (WSN−) were stimulated with human IL-28A (B) or IL-29 (50 ng/ml) (C) for indicated time. Cell lysates were analyzed by Western blotting using indicated antibodies. (**D**) Levels of phosphorylated STAT1 in (C) were quantitated by densitometry, and normalized to STAT1 expression and control β-actin levels. In each experiment, the highest level of STAT1 phosphorylation is 100. Plotted are the average levels from three independent experiments. The error bars represent the S.E. (**E**) A549 cells were infected with WSN (MOI = 1), lysed at the 0, 5, 10, 15 and 20 h p.i., and analyzed by Western blotting using indicated antibodies. (**F**) A549 cells were either stimulated by supernatant (SN) culture medium from IAV-infected cells in (E) or infected with WSN for 1 h, followed by Western blotting with indicated antibodies.

In normal cells, the strength and duration of cytokine signaling are tightly regulated. However, little is known about why a huge amount of IFN-λ is induced during IAV infection. To address this issue, we sought to investigate whether regulation of IFN-λ-mediated signaling was altered during the viral infection. IFN-λ, like type I IFN, primarily activates the JAK-STAT signal pathway to achieve its antiviral function. Unlike type I IFN receptor, IFN-λ receptor is expressed in a cell-specific fashion [28]. Here we observed that IFN-λ was able to activate JAK-STAT signal pathway in A549 cells (Figure 2A, B). Furthermore, the level of IFN-λ-induced STAT1 phosphorylation was markedly reduced in IAV infected cells, as compared with that in control cells (Figure 2B–D). To substantiate this finding, a time course experiment was performed. We found that phosphorylation of STAT1 in infected cells was dramatically inhibited at later stages of infection (after 15 h p.i.) (Figure 2E), while no significant decrease in STAT1 phosphorylation was observed in the cells treated with corresponding culture supernatants (SN) from the infected cells (Figure 2F). These data indicate that activation of JAK-STAT signaling by IFN-λ was suppressed in the presence of IAV.

### Intracellular detection of IAV infection induces robust expression of SOCS-1, leading to inhibition of STAT1 activation

**Figure 3:**
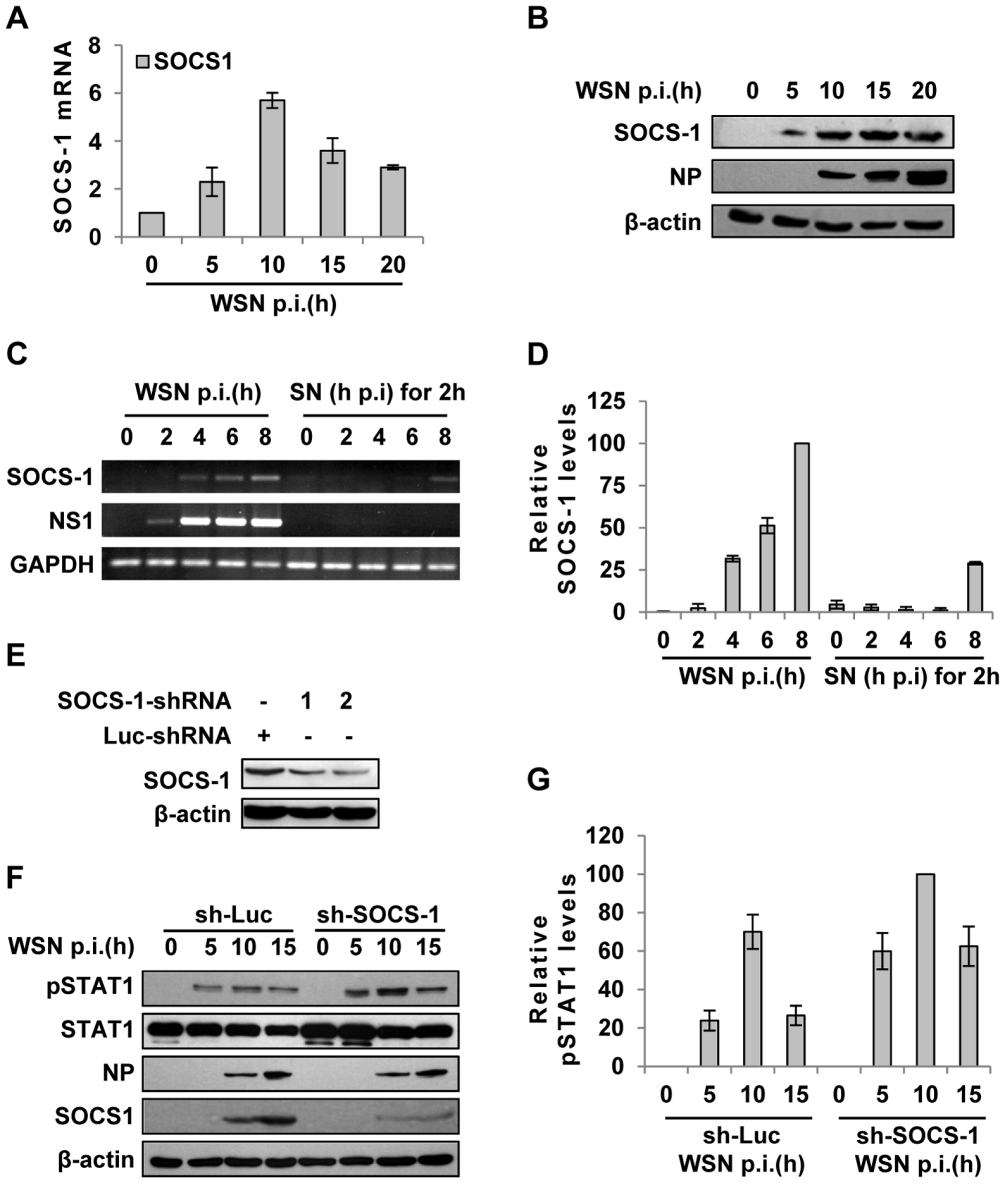
IAV infection induces robust expression of SOCS-1, resulting in decreased phosphorylation of STAT1. (**A**) Quantitative real-time RT-PCR was performed to examine the expression of SOCS-1 in A549 infected with WSN for indicated time. (**B**) Lysates from cells in (A) were analyzed for the protein levels of SOCS-1, as detected by Western blotting with indicated antibodies. (**C**) A549 cells were infected by WSN for indicated time. Supernatants (SN) derived from these cells were used to stimulate the native A549 for 2 h. Both infected cells and supernatants-stimulated cells were lysed and analyzed for SOCS-1 expression by RT-PCR. (**D**) SOCS-1 levels in (C) were quantitated by densitometry, and normalized to GAPDH levels as described in Figure 2D. Plotted are the average levels from three independent experiments. The error bars represent the S.E. (**E**) A549 cells expressing shRNAs targeting either SOCS-1 or control luciferase (Luc) were infected with WSN for 15 h. Western blotting was performed to determine the interference efficiency. Treatment with SOCS-1-shRNA#2 caused approximately 75% reduction in SOCS-1 expression quantitated by densitometry. Thus, SOCS-1-shRNA#2 was used in this study. (**F**) SOCS-1-ablated or control A549 cells were infected with WSN for the indicated time. Cell lysates were analyzed by Western blot probed with the antibodies as indicated. (**G**) Levels of phosphorylated STAT1 in (F) were quantitated by densitometry, and normalized to control β-actin levels as described in Figure 2D. Plotted are the average levels from three independent experiments. The error bars represent the S.E.

Next, we further investigated how IAV infection inhibits IFN-λ-induced STAT1 phosphorylation in A549 cells. Of the eight members of SOCS family, SOCS-1 is the most potent inhibitor of cytokine-induced signaling. In addition, it has recently emerged that SOCS-1 is an important regulator of innate immune response triggered by IAV [18]. Therefore, we hypothesized that SOCS-1 is involved in inhibition of STAT1 phosphorylation during IAV infection. To test this, SOCS-1 mRNA levels in A549 cells during IAV infection were examined by quantitative RT-PCR (Figure 3A). The mRNA level of SOCS-1 was significantly up-regulated at early stages and began to reduce at late stages of infection, but its protein level was consistently increased (Figure 3B). Immunofluorescence study showed that increased expression of SOCS-1 and inhibition of STAT1 phosphorylation occurred specifically in IAV infected cells (Figure S2A, S2B). This implies that there might be a certain relationship between expression of SOCS-1 protein and inhibition of STAT1 phosphorylation. Surprisingly, although SOCS-1 expression in A549 cells was induced by supernatants derived from infected cell culture at later stages (Figure 3C, D and Figure S2C), the SOCS-1 expression induced by IAV infection appeared earlier than that triggered by cell culture supernatants (Figure 3C, D), and than the initial production of IL-29 protein (Figure S2D). The results strongly suggest that during IAV infection, there was a cytokine-independent mechanism to induce SOCS-1 expression.

To further verify the functional involvement of SOCS-1 in the suppression of STAT1 activation, SOCS-1 expression in A549 cells was knocked down by shRNA (Figure 3E). In SOCS-1 knockdown A549 cells, but not the control cells, the level of STAT1 phosphorylation was notably increased during the infection (Figure 3F, G), indicating that SOCS-1 was a direct inhibitor of STAT1 phosphorylation during IAV infection.

### SOCS-1-mediated inhibition of cytokine signaling contributes to excessive production of IFN-λ during IAV infection

**Figure 4:**
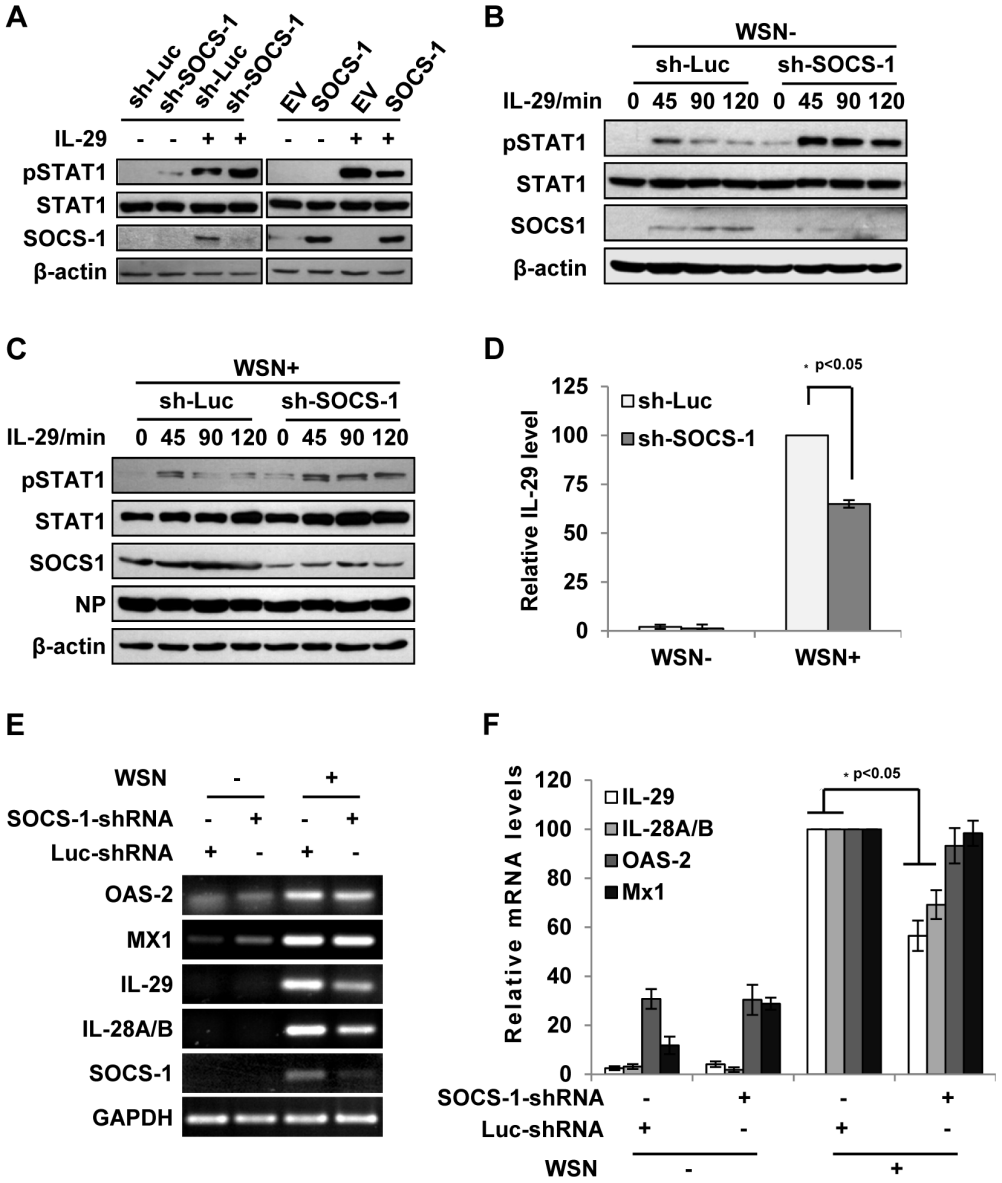
Inhibition of cytokine-mediated STAT1 activation by SOCS-1 contributes to overproduction of IFN-λ during IAV infection. (**A**) A549 cells expressing SOCS-1, empty vector (EV) or shRNAs targeting SOCS-1 or luciferase (Luc) were treated with or without IL-29 (50 ng/ml) for 45 min. Cell lysates were analyzed by Western blotting using indicated antibodies. (**B, C**) Luc or SOCS-1 knockdown A549 cells were infected without (B) or with (C) WSN virus for 15 h and then treated with IL-29 for indicated time. Shown are immunoblots of the cell lysates probed with indicated antibodies. (**D, E**) SOCS-1-ablated or control A549 cells were infected with or without WSN virus for 15 h. Subsequently, IL-29 levels in the supernatants from the cell culture were examined by ELISA. IL-29 levels produced by infected control cells were set to 100%. Plotted are the average results from three independent experiments and the error bars represent the S.E. (D). mRNA levels of OAS-2, Mx1, IL-28A/B, and IL-29 were examined by RT-PCR (E). (**F**) Levels of these mRNAs in (E) were quantitated by densitometry, and normalized to GAPDH levels as described in Figure 2D. mRNA level in infected control cells is 100. Plotted are the average results from three independent experiments. The error bars represent the S.E. Statistical significance of change was determined by Student's t-test (*P<0.05).

Since JAK-STAT signaling was inhibited by IAV-induced SOCS-1, we asked whether the activated JAK-STAT pathway by IFN-λ is also disrupted by SOCS-1. To address this issue, we examined the effect of SOCS-1 protein on activation of STAT1 by IL-29. As shown in Figure 4A, down-regulation of SOCS-1 enhanced IL-29-induced activation of STAT1, whereas overexpression of SOCS-1 inhibited IL-29-induced activation of STAT1 in A549 cells, revealing that SOCS-1 negatively regulates IL-29-mediated STAT1 signaling. Moreover, in IAV-infected SOCS-1-ablated A549 cells, STAT1 phosphorylation was markedly elevated by IL-29 stimulation, and this effect was prolonged in both infected and uninfected cells when compared to the control cells (Figure 4B, C). These findings suggest that SOCS-1 inhibits IL-29 signal pathway during IAV infection.

Since IAV-induced expression of SOCS-1 appeared earlier than expression of IFN-λ (see above description), it was interesting to investigate whether the induced SOCS-1 influenced IFN-λ production. Surprisingly, the protein level of IL-29 was significantly reduced in the SOCS-1-ablated cells, comparing with that in the control cells infected with IAV (Figure 4D). Furthermore, the mRNA levels of IL-29 and IL-28A/B were also significantly reduced in SOCS-1-ablated A549 cells (Figure 4E, F), while the mRNA levels of ISGs (MX1 and OAS-2) did not change significantly at this time point (Figure 4E, F). The results suggest that the antiviral response is not affected despite less production of IFN-λ in SOCS-1-ablated cells.

### Forced activation of STAT1 causes a significant decrease in type III IFN expression during IAV infection

**Figure 5:**
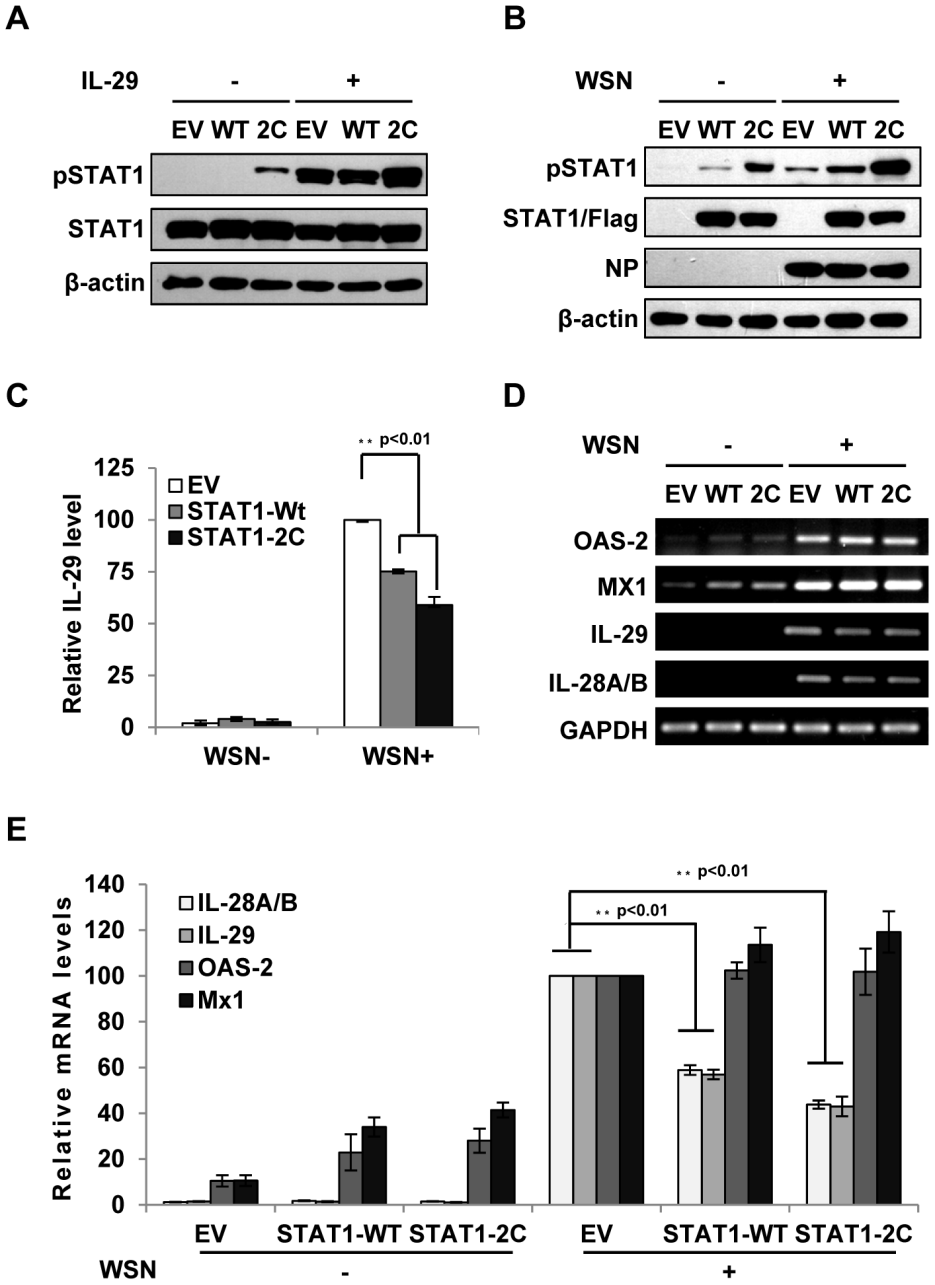
Forced activation of STAT1 causes a significant decrease in IFN-λ expression during IAV infection. (**A**) A549 cell lines stably expressing STAT1-WT, STAT1-2C or empty vector (EV) were treated with or without IL-29 (50 ng/ml) for 45 min. Cell lysates were analyzed by Western blot using indicated antibodies. (**B–D**) A549 cell lines described in (A) were infected with or without WSN virus for 15 h. Subsequently, the cell lysates were analyzed by Western blot probed with indicated antibodies (B), and the protein levels of IL-29 in the cell culture supernatants were examined by ELISA (C). IL-29 levels produced by infected cells expressing EV were set to 100%. Plotted are the average results from three independent experiments. The error bars represent the S.E. mRNA levels of OAS-2, Mx1, IL-28A/B and IL-29 were measured by RT-PCR (D). (**E**) IFN-λ levels and OAS-2 and Mx1 levels in (D) were quantitated by densitometry, and normalized to GAPDH levels as described in Figure 2D. Plotted are the average levels from three independent experiments. The error bars represent the S.E. Statistical significance of change was determined by Student's t-test (*P<0.05, **P<0.01).

Because the results presented above revealed that IFN-λ-mediated activation of STAT1 was abrogated during IAV infection, next we asked whether forced activation of STAT1 had any effect on expression of IFN-λs. To test this possibility, we generated A549 cell lines stably expressing either empty vector (EV), STAT1 wild type (WT), or constitutively activated form STAT1-2C (2C) [29], [30]. The enhancement of STAT1 phosphorylation during IAV infection or stimulated by IFN-λ was confirmed in STAT1-2C-expressing cells (Figure 5A, B). STAT1 phosphorylation was also increased in infected cells overexpressing STAT1-WT as compared to the control cells (Figure 5B). Interestingly, production of IL-29 protein was remarkably decreased in the STAT1-2C-expressing cells as compared to the control after IAV infection (Figure 5C). Consistent with this observation, the mRNA levels of IL-29 and IL-28A/B were significantly reduced in IAV infected STAT1-2C-expressing cells (Figure 5D, E). Furthermore, we tested whether alteration of IFN signaling had any effect on IFN-α and IFN-β production. We found that silencing SOCS-1 or overexpression of STAT1 slightly reduced the type I IFN production during IAV infection (Figure S3A–C). On the other hand, no significant change in the induction of OAS-2 and Mx1 was observed in these cells at late time points post infection (Figure 5D, E). Interestingly, at early time point post infection, activation of STAT1 signaling promoted expression of OAS-2 and Mx1 (Figure S3D–F).

### Disruption of IFN-λ signaling pathway results in robust activation of NF-κB during IAV infection

In an attempt to provide insights into the mechanism of how inhibition of cytokine signaling causes excessive expression of IFN-λ during IAV infection, we evaluated the pathway governing IFN-λ expression. We found that level of viral RNA, the inducer of IFN-λ expression was unchanged by silencing SOCS-1 expression or forcing STAT1 activation (Figure S3G, S3H). Furthermore, forced activation of cytokine signaling did not alter expression of Pattern-Recognition Receptors (PRRs) including RIG-I and TLR3 (Figure S3H). Expression of TLR-7/8 was also examined but they were undetectable in A549 cells.

**Figure 6:**
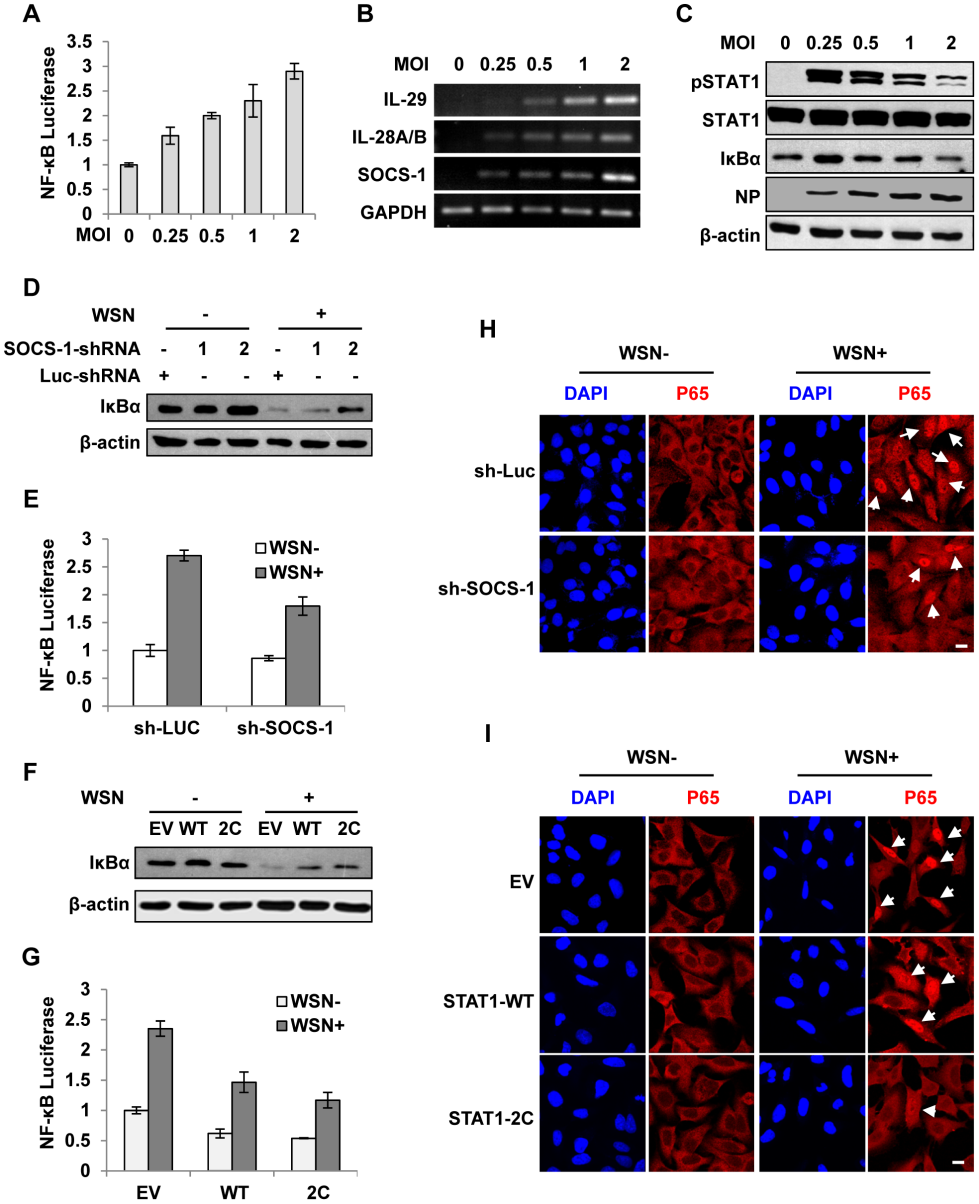
Disruption of cytokine signaling pathway results in robust activation of NF-κB during IAV infection. (**A**) 293T cells were co-transfected with pNF-κB-Luc and pRL-TK for 10 h and then infected with WSN virus at indicated MOI for 15 h. Luciferase activity in cell lysates was measured and displayed as the mean ± SD of relative luciferase units normalized to Renilla luciferase activity from three independent experiments. (**B, C**) A549 cells were infected with WSN virus as described in (A). RT-PCR was performed to examine the expression of indicated genes (B), and Western blotting was performed using indicated antibodies (C). (**D**) A549 cells expressing shRNAs targeting SOCS-1 or luciferase were infected with or without WSN virus for 15 h, followed by Western blotting with indicated antibodies. (**E**) 293T cells were co-transfected with pNF-κB-Luc, pRL-TK and either SOCS-1 shRNA expressing vector or control for 10 h and then infected with WSN virus for 15 h. Luciferase activity was analyzed as described in (A). (**F**) A549 cells expressing STAT1-WT, STAT1-2C or control were infected with or without WSN virus and analyzed by Western blotting with indicated antibodies. (**G**) Experiments were carried out as described in (E). Shown are results from experiments using cells expressing STAT1-WT, STAT1-2C or control. (**H**) A549 cells stably expressing SOCS-1 shRNA or control were infected with or without WSN virus for 15 h. Immunofluorescence staining was performed using an anti-p65 antibody to detect translocation of NF-κB. The nuclei were stained with DAPI. Bar, 10 µm. (**I**) Experiments were carried out as described in (H). Shown are results from experiments using cells expressing STAT1-WT, STAT1-2C or control. Bar, 10 µm.

Since alteration of cytokine signaling did not affect the levels of PRRs and viral RNA and given that IRF3 is a known regulator of IFN expression at the early stage of infection, we determined whether there was a functional link between IFN-λ signaling and activation of nuclear factor of κB (NF-κB), a key transcriptional factor downstream of RIG-I pathway [31]. To this end, cells were infected with IAV using increasing MOI. Interestingly, experiment using luciferase reporter gene revealed a positive correlation between increased NF-κB activity and increased expression of SOCS-1 and IFN-λ in infected cells (Figure 6A, B). By contrast, STAT1 phosphorylation and IκB protein levels were consistently reduced (Figure 6C). To further confirm this finding, we employed the A549 cell lines stably expressing SOCS-1 shRNA or active form of STAT1. We observed that in infected cells, depletion of SOCS-1 increased IκB protein level (Figure 6D) and significantly decreased NF-κB activation (Figure 6E). Similarly, forced activation of STAT1 inhibited the degradation of IκB (Figure 6F), and as a result, activation of NF-κB was significantly suppressed in the infected cells (Figure 6G). In contrast, low IκB level and high level of NF-κB activation were detected in SOCS-1-overexpressing cells after IAV infection even using low MOI (Figure S4A, S4B). Consistent with these observations, immunofluorescence microscopy study showed that nuclear translocation of NF-κB p65 was significantly abrogated in SOCS-1-ablated or STAT1-activated cells infected with IAV (Figure 6H, I and Figure S4C, S4D). Together, these results suggest that disruption of cytokine signaling pathway results in robust activation of NF-κB, which causes excessive production of IFN-λ during IAV infection.

### Suppression of cytokine signaling by IAV-induced SOCS-1 contributes to overproduction of IFN-λ *in vivo*

**Figure 7:**
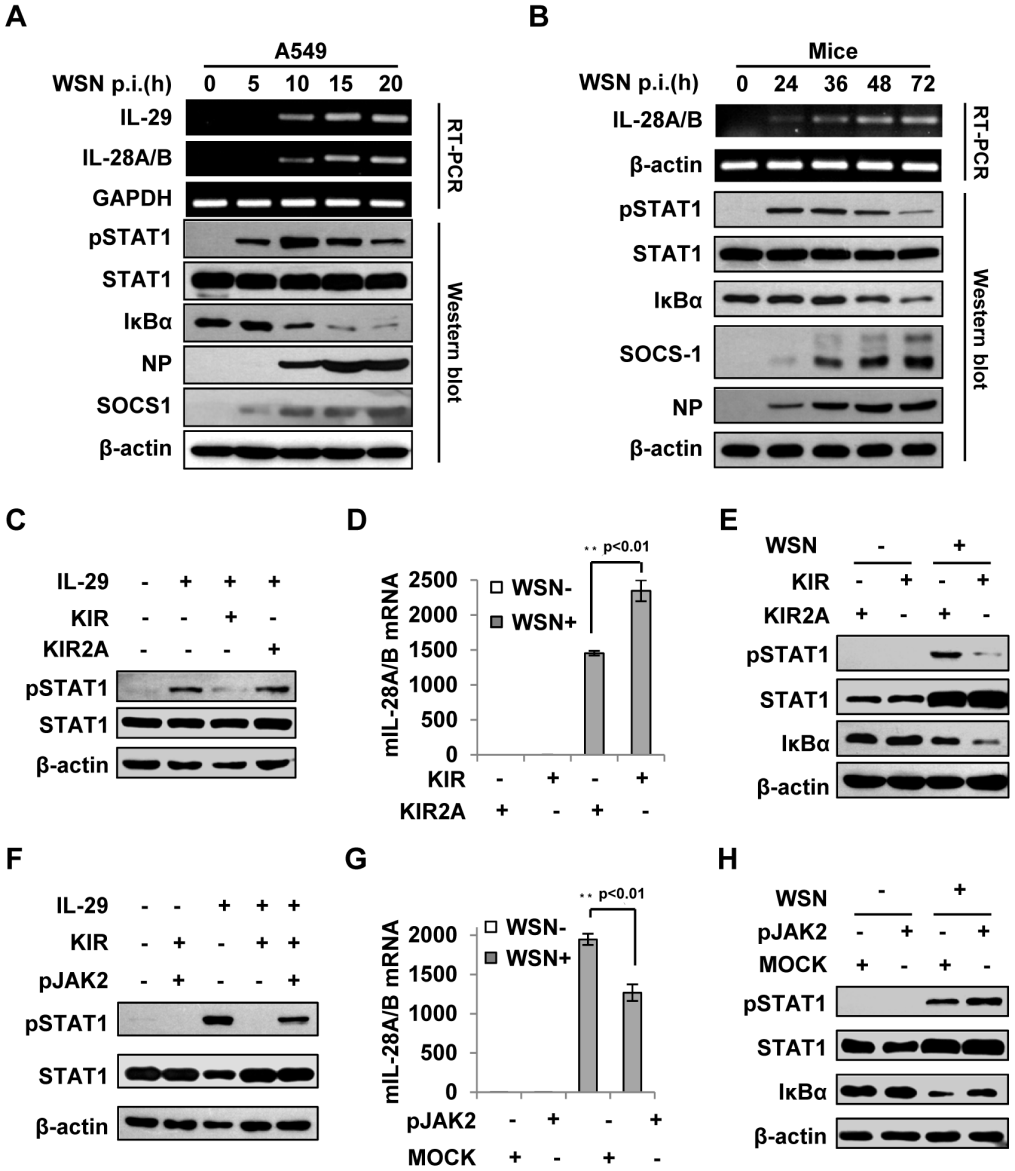
Suppression of cytokine signaling by SOCS-1 contributes to overproduction of IFN-λ in mice. (**A**) A549 cells were infected with WSN for indicated time. Subsequently, the cell lysates were analyzed by Western blot probed with indicated antibodies, and the mRNA levels of IL-28A/B and IL-29 were measured by RT-PCR. (**B**) BALB/c mice were infected intranasally with or without WSN virus (1×10^5^ PFU) for indicated time. The lungs were lysed and analyzed by Western blot probed with indicated antibodies. Expression of IL-28A/B in lung was measured by RT-PCR. (**C**) A549 cells were treated with or without 10 µM lipo-SOCS-1-KIR or lipo-SOCS-1-KIR2A for 20 min and then stimulated with or without IL-29 for 45 min, followed by Western blotting using indicated antibodies. (**D, E**) Mice were treated twice with lipo-SOCS-1-KIR peptide or lipo-SOCS-1-KIR2A control peptide at 5 µg/g body weight by intraperitoneal injection (i.p.) and then inoculated intranasally with or without WSN (1×10^5^ PFU). On Day 3 p.i., expression of IL-28A/B in lung was examined by real-time PCR (D), and Western blotting was performed using antibodies as indicated (E). (**F**) A549 cells were treated with 10 µM lipo-SOCS-1-KIR, lipo-pJAK2 or both and then stimulated with IL-29 as described in (C). Shown is a Western blot probed with indicated antibodies. (**G–H**) Mice were treated with or without lipo-pJAK2 peptide at 5 µg/g body weight and then infected with or without WSN as described in (D). IL-28A/B expression was examined by real-time PCR (G), and immunoblot was probed as indicated (H).

To confirm the correlation of type III IFN expression with the activation of STAT-1 and NF-κB signaling, a time course study was performed in more detail in infected cell culture (Figure 7A). The results indicated that disruption of IFN-λ signal by SOCS-1 increased their expression likely through activating NF-κB in IAV infected cells. Next, we sought to determine the expression levels of SOCS-1 in mouse lung at different stages of IAV infection. We found that MLD50 of the WSN virus was approximately 3×10^3^ pfu under our conditions, consistent with the previous observation [32]. Therefore, mice were inoculated intranasally with 1×10^5^ pfu of the virus (about 33 MLD50) as previously described [33], [34]. As shown in Figure 7B, expression of SOCS-1 protein was consistently increased during 3 days of infection. As a consequence, STAT1 phosphorylation was inhibited (Figure 7B). Moreover, activity of NF-κB was elevated during the infection as indicated by the gradually diminished IκBα levels, suggesting that the expression kinetics of IFN-λ correlated with NF-κB activation. Of interest, expression of SOCS-1 was earlier and faster than that of IFN-λ (Figure S5A, B), suggesting that SOCS-1 expression is cytokine-independent at least at the early stage of infection and SOCS-1 might regulate IFN-λ expression beyond negative feedback regulation to respond the cytokines *in vivo*. This finding is consistent with our *in vitro* results presented above (Figure 3C, D).

To further address the relationship between the expression of SOCS-1 and the induction of IFN-λ, membrane-permeable peptides of SOCS-1-KIR and pJAK2 were used to mimic SOCS-1 overexpression and counteract SOCS-1 function, respectively. The functions of these peptides in IFN-λ response were confirmed in *vitro*, as the phosphorylation of STAT1 stimulated by IL-29 was dramatically inhibited in the presence of SOCS-1-KIR but not the control peptide SOCS-1-KIR2A (Figure 7C), and the inhibitory effect of SOCS-1-KIR on STAT1 phosphorylation was markedly diminished when SOCS-1-KIR was added together with pJAK2 peptide (Figure 7F). When mice were treated with these peptides and then inoculated with IAV, IFN-λ level was significantly increased in mice treated by SOCS-1-KIR (Figure 7D and Figure S5C). By contrast, the expression of IFN-λ in mice treated with pJAK2 peptide was significantly reduced as compared to control group (Figure 7G and Figure S5D). Furthermore, low levels of STAT1 phosphorylation and IκBα protein were found in SOCS-1-KIR treated mice after IAV infection (Figure 7E), whereas high levels of STAT1 phosphorylation and IκBα protein were present in pJAK2 treated group (Figure 7H). In addition, our experiments showed that treatment with SOCS-1-KIR increased mouse body weight loss, whereas pJAK2 treatment reduced the body weight loss during IAV infection (Figure S5E–F). Together, these data suggest that JAK-STAT signaling pathway is disrupted by increased SOCS-1 in infected mice, which results in an increase in IFN-λ expression likely through activating NF-κB.

### Silencing SOCS-1 significantly reduces IFN-λ expression in transgenic mice during IAV infection

**Figure 8:**
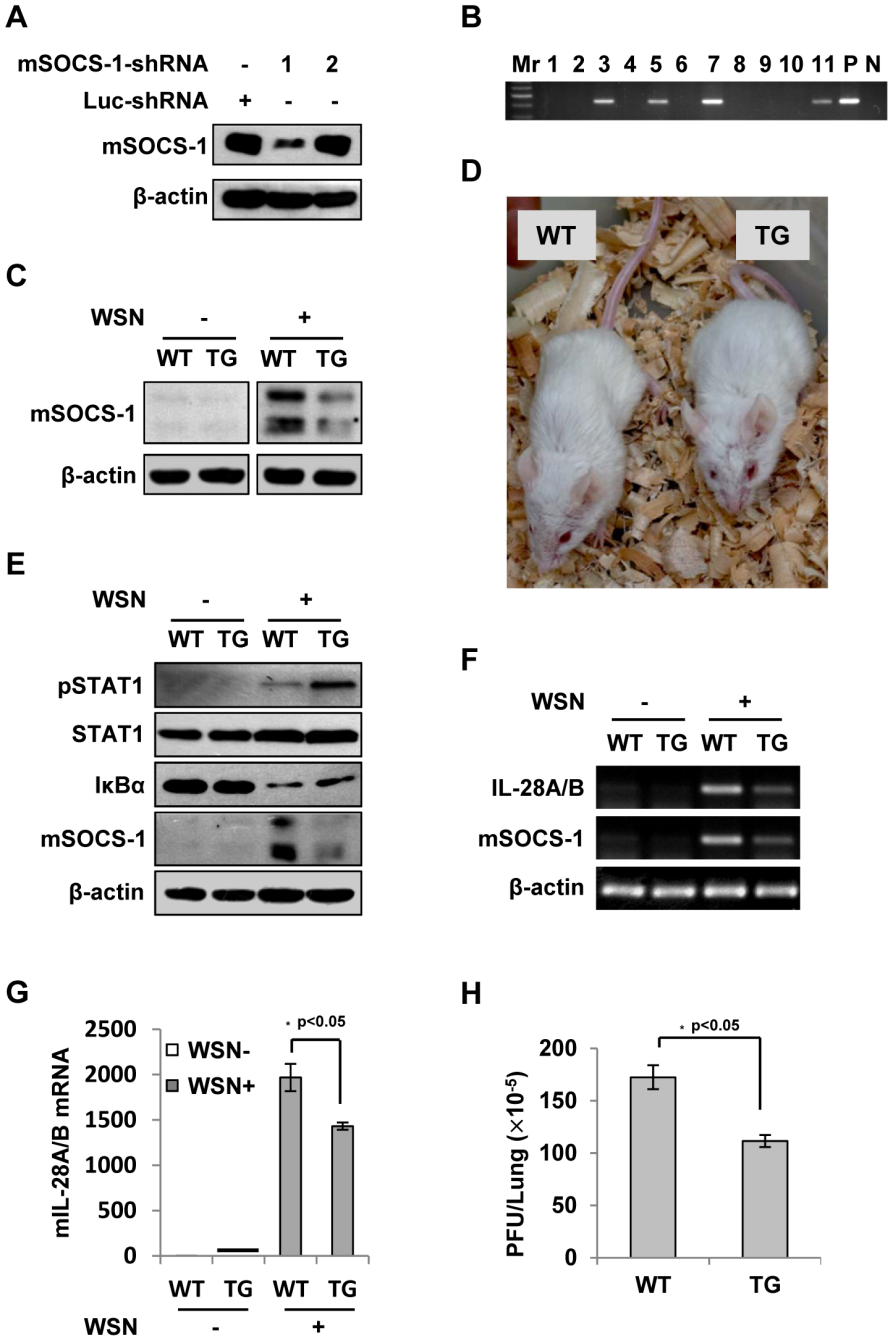
Silencing SOCS-1 causes a significant reduction of IFN-λ expression in transgenic mice during IAV infection. (**A**) Immunoblotting was performed to test shRNA-based knockdown of mouse SOCS-1 in transfected cell line. (**B**) The SOCS-1-knockdown transgenic mice were genotyped by PCR. Shown is representative genotyping of SOCS-1-knockdown transgenic mice. Numbers 1-11, representative transgenic mice and wild type littermates; P, positive control; N, negative control. (**C**) SOCS-1 expression in representative tissues (lung) from SOCS-1-knockdown transgenic mice (TG) and wild-type littermates (WT) was examined by immunoblotting after WSN infection. (**D**) The transgenic founders with high interference efficiency were selected and maintained on a BALB/c genetic background. Shown is a representative photograph of SOCS-1-knockdown transgenic mouse and wild-type littermate. (**E–H**) WT and TG mice were infected with or without WSN virus as described in Figure 7. On Day 3 p.i., lungs were lysed and analyzed by Western blotting with indicated antibodies (E), IL-28A/B expression was examined by RT-PCR (F) and real-time PCR (G), and viral titers in lungs of WT and TG mice were examined by plaque assay and values are shown as mean ± SD (H).

To further define the role of SOCS-1 in IFN-λ production induced by IAV, we wished to establish a more physiological model system for analysis of SOCS-1 involvement in this process. For this, SOCS-1-knockdown transgenic mice (TG) were generated as previously described (Figure 8A–D) [35], [36]. The transgenic founders with high interference efficiency were selected (Figure 8C, D). We found that the level of STAT1 phosphorylation was greatly increased in TG compared to wild type (WT) mice after IAV infection (Figure 8E). In contrast, the activity of NF-κB was reduced as indicated by increased IκBα level. Consistent with this, expression of IFN-λ was significantly decreased in IAV infected TG mice (Figure 8F, G). Furthermore, by haematoxylin and eosin (HE) staining, we found that on Day 3 p.i., the lung in mice showed obvious inflammation, but the inflammation in the lung of SOCS-1 knockdown TG mice was minor compared to WT control (Figure S6A). Less body weight loss was observed and Less viral load was detected in the lung of TG mice than that in WT group (Figure 8H and Figure S6B–C), suggesting that although IFN-λ expression is reduced in SOCS-1 knockdown TG mice, the innate antiviral immune response is enhanced.

## Discussion

The clearance of IAV during infection depends on the activation of effective innate and adaptive immune responses. Cytokines activate innate immune responses and initiate the development of adaptive, virus-specific immune responses [37], [38]. Thus, cytokines play critical roles in defense against the virus infection. Various types of cells in host secrete cytokines and chemokines following IAV infection. Among these cell types, epithelial cells are thought to be one of the most important cytokine-producing cells during IAV infection, and believed to be vital for the virus-induced cytokine storm [39]. We have previously profiled the cellular transcriptional response to IAV infection in human type II alveolar epithelial cell line A549 and found that this type of cell expresses many different cytokines and chemokines after the virus infection [22]. In this study, we show that IAV infection induces excessive expression of IFN-λ that is mainly dependent on RIG-I signaling and partially on TLR3 signaling, indicating that they are involved in the innate antiviral response to the infection. This observation is consistent with previous studies showing that IFN-λ are the predominant IFNs induced by respiratory viruses and have a wide range of antiviral functions in response to respiratory viruses [11], hepatitis C virus [40], rotavirus [12], herpes simplex virus [41] and influenza virus [42].

IFN-λ receptor complex is composed of the ubiquitously expressed short chain IL-10R2 and the long chain IL-28R1 expressed preferentially on epithelial cells [12]. IFN-λs bind the receptors to activate the JAK-STAT signaling pathway which initiates transcription of the ISGs. Thus, IFN-λs, like other types of IFNs, play important roles in the control of IAV propagation in epithelial cells [42], [43]. Since IFNs are important in a variety of cellular processes, their production and response are delicately regulated by multiple mechanisms. Viruses have evolved different ways to counteract these mechanisms, leading to dysregulation of IFN expression and function, and then successfully evaded the host antiviral response. IAV also exerts its effects through some mechanisms. For example, the viral non-structural protein 1 (NS1) has been shown to inhibit type I IFN response and block IFN-β production. On the other hand, it has also been shown that the capacity of NS1 to confer resistance to host immune response by decreasing sensitivity to particular cytokines causes their overproduction [39]. It remains an ongoing task to determine whether overproduction of IFN-λs is regulated by the NS1.

Recently, it has been shown that IAV induces expression of SOCS-1 and SOCS-3 to negatively regulate JAK-STAT pathway and thereby down-regulates the innate immune response including abrogation of the type I IFN signaling [17]. In the present study, we found that SOCS-1 was greatly induced before the abundant secretion of cytokines in both IAV-infected A549 cells and IAV-infected mice, strongly indicating that during IAV infection, there is a cytokine-independent mechanism to provoke SOCS-1 expression at least at the early stage. Similarly, it has been reported that early induction of SOCS-3 transcription is IFN-β-independent [17]. However, Julien and coworkers have observed that up-regulation of SOCS-1 and SOCS-3 in IAV-infected cells is IFNAR1-dependent [18], which does not contradict with our observation, because we found that the culture supernatants at the later stages of infection indeed stimulated SOCS-1 expression. Therefore, we conclude that IAV might induce cytokines-independent SOCS-1 expression through other mechanisms, and at least this is true at the early stage of IAV infection.

Our experiments demonstrated that IAV-provoked STAT1 phosphorylation at the early stage of infection was inhibited by the virus-induced SOCS-1. Furthermore, we provided evidence that JAK-STAT signaling activated by IFN-λ was also inhibited by SOCS-1. It has been previously shown that IAV abrogates the innate immune response mediated by type I IFNs and IFN-γ by disruption of the JAK-STAT signaling pathway [17], [20]. However, little is known about how suppression of cytokine signaling by SOCS proteins affects the production of IFNs during IAV infection. Interestingly, here we found that the IFN-λ levels were significantly decreased in IAV infected SOCS-1-depleted A549 cells and transgenic mice as compared to infected controls. Importantly, forced activation of STAT1 also significantly inhibits the production of IFN-λ *in vitro* and *in vivo*. Despite decreased expression of IFN-λ, the antiviral response was not impaired in SOCS-1-depleted cell and animal. These results suggest that suppression of IFN-λ signaling by SOCS-1 results in their excessive production during IAV infection. Our hypothesis is that suppression of cytokine signaling by virus-induced SOCS-1 leads to an adaptive increase in IFN-λ production by host to protect cells against viral infection. However, increased IFN-λ further induces the expression of SOCS-1 at late stage of infection, which in turn, inhibits the activation of JAK-STAT signaling. Finally, this vicious cycle results in the excessive production of IFN-λ with an impaired antiviral activity due to increased SOCS-1 protein during IAV infection. Although we observed that forced activation of IFN signal also slightly decreased the levels of type I IFNs, whether this hypothesis applies to other cytokine storm provoked by highly virulent influenza virus infection is unclear. In addition, we found that after IAV infection, the SOCS-1 knockdown transgenic mice did not display a remarkable phenotype as compared to wild type mice. However, it is possible that SOCS-1-mediated upregulation of IFN-λ levels has a more prominent role in pathogenesis of highly pathogenic strains of IAV that elicit hypercytokinemia and lethal phenotypes. These remain to be further determined.

Our study has also begun to address the mechanism by which inhibition of cytokine signaling causes the excessive expression of IFN-λ during IAV infection. We presume that the repression of STAT1 might activate other transcriptional factors to elevate cytokine levels. Previous studies have shown that aryl hydrocarbon receptor couples with STAT1 to regulate lipopolysaccharide-induced inflammatory responses [44]. It has also been revealed that progressive dysregulation of NF-κB and STAT1 leads to pro-angiogenic production of CXC chemokines [45]. It is thought that NF-κB and STAT1 might have a crosstalk [46]–[48]. Although it is unclear how the phosphorylation of STAT1 can be associated with NF-κB activation [49], our data showed that IAV inhibited STAT1 activation but promoted the degradation of IκBα, and thus the activity of NF-κB was enhanced both *in vitro* and *in vivo*. Moreover, our results revealed that IκBα was degraded and thereby the activity of NF-κB was increased when SOCS-1 was up-regulated by IAV. In fact, this finding is consistent not only with the enhancement of NF-κB activity in SOCS-1 overexpressed-keratinocytes after stimulation by the poly-(I∶C), but also with the increased NF-κB activation in SOCS-1-transfected cells [18], [50]. Together, these data suggest that suppression of cytokine signaling by SOCS-1 may influence the NF-κB activation. Further research is required to address how inhibition of JAK-STAT signaling is involved in regulation of NF-κB activation.

## Materials and Methods

### Ethics statement

The mouse experimental design and protocols used in this study were approved by “the regulation of the Institute of Microbiology, Chinese Academy of Sciences of Research Ethics Committee” (Permit Number: PZIMCAS2012001). All mouse experimental procedures were performed in accordance with the Regulations for the Administration of Affairs Concerning Experimental Animals approved by the State Council of People's Republic of China.

### Influenza virus and infection

Influenza virus strain A/WSN/33 (H1N1) was prepared as previously described [22], [51]. For infection, cells were washed with phosphate-buffered saline (PBS) and infected with the multiplicity of infection (MOI) as indicated in the figure legends. After adsorption with α-MEM medium containing 2 µg/ml TPCK (L-1-tosylamido-2-phenylethyl chloromethyl ketone)-treated trypsin, 100 U/ml penicillin, and 100 µg/ml streptomycin for 45 minutes at 37°C, the supernatant was aspirated and cells were cultured with the α-MEM medium for indicated time. To inactivate the viruses, equal amounts of viruses were incubated at 56°C or 65°C for 30 minutes as described previously [24].

### Plasmids and luciferase assay

Plasmids pRC-CMV-STAT1-WT and pRC-CMV-STAT1-2C in which N658 and A656 of STAT1 were substituted by cysteine residues were kindly provided by Dr. David A. Frank (Dana-Farber Cancer Institute, Boston, MA). The cDNA coding STAT1-WT or STAT1-2C was subcloned into the *Not* I/*Sal* I sites of retroviral vector pMSCV-IRES-GFP (pMIG) to generate pMIG-STAT1-WT and pMIG-STAT1-2C. The vector pMIG-SOCS-1 was previously described [13]. NF-κB-luciferase reporter named pNF-κB-Luc and Renilla luciferase reporter named pRL-TK were gifts from Dr. Shijuan Gao (Institute of Microbiology, Chinese Academy of Sciences). For luciferase assay, cells were co-transfected with pNF-κB-Luc, pRL-TK and indicated plasmids, and luciferase activity was measured using the dual-luciferase reporter assay system according to the manufacturer's instruction (Promega, U.S.).

### Antibodies and peptides

The following antibodies were used in this study: anti-STAT1 (E23), anti-phospho-STAT1 (Tyr701), anti-RIG-I, anti-NF-κB p65 (Santa Cruz Biotechnology, Santa Cruz, CA); and anti-β-actin (Abcam). All other antibodies were obtained as previously described [13], [51]. Peptides of SOCS-1-KIR (^(53)^DTHFRTFRSHSDYRRI), SOCS-1-KIR2A (^(53)^DTHF**A**TF**A**SHSDYRRI) and pJAK2 (^(1001)^LPQDKE**Y**YKVKEP) were synthesized by ChinaPeptides (Shanghai, China). All peptides were synthesized with an attached lipophilic group, palmitic acid, to facilitate entry into cells as previously described [16], [52]. Peptides were purified by preparative RP-HPLC, and were characterized by LC-MS and HPLC analysis.

### Cytokines, cell stimulation, and ELISA

Recombinant human IL-28A and IL-29 were purchased from PeproTech (Rocky Hill, NJ). Cells were incubated with the recombinant IL-29 (50 ng/ml) for 45 minutes for stimulation, unless otherwise indicated. Supernatant culture medium from the A549 cells infected with IAV strain A/WSN/33 (H1N1) was also used as a source of virus-induced cytokines for cell stimulation. To quantify IL-29 production by host cells, supernatant culture medium from virus infected cells was harvested and examined by enzyme-linked immunosorbent assay (ELISA) using the ready-SET-Go of human IL-29 analysis kit (eBioscience, San Diego, CA) according to manufacturer's instruction.

### Stimulation of cells with RNA

Total RNA was prepared from A549 cells infected with the IAV for 8 hours (viral RNA) or from uninfected cells (cellular RNA) using Trizol (TIANGEN BIOTECH BEIJING CO., LTD.) according to manufacturer's instructions. The calf intestine alkaline phosphatase (CIAP) (TaKaRa) was used to dephosphorylate viral 5′-triphosphate RNA as previously described [17]. A549 cells were transfected with the isolated RNA using Lipofectamine 2000 (Invitrogen). Supernatant medium from transfected cells was harvested and examined by ELISA for IL-29 production. The transfected cells were lysed and examined by real-time PCR for expression of indicated genes.

### Western blotting and immunofluorescence

For Western blotting analysis, cell lysates were separated by SDS-polyacrylamide gel electrophoresis, transferred onto a nitrocellulose membrane, and probed with indicated antibodies as described previously [53]. To detect nuclear translocation of NF-κB p65, immunofluorescence was performed as described previously [22]. Images were acquired using a confocal microscope (Model LSCMFV500) and a 60× oil immersion objective lens (both from Olympus Optical, Japan) with an NA of 1.40.

### RNA interference and generation of stable cell lines

Short hairpin RNA (shRNA)-based knockdown cell lines were generated by infection of A549 with lentiviruses expressing specific shRNA in pSIH-H1-GFP vector as described previously [22]. The sequences used in the shRNAs targeting specific genes were as follows: human SOCS-1 shRNA#1 5′-GCATCCGCGTGCACTTTCA-3′ [54] and shRNA#2 5′-CTACCTGAGCTCCTTCCCCTT-3′ [55], mouse SOCS-1 shRNA#1 5′-GGACGCCTGCGGCTTCTAT-3′ and shRNA#2 5′-CTACCTGAGTTCCTTCCCCTT-3′ [56], human RIG-I shRNA#1 5′-TGCAATCTTGTCATCCTTTAT-3′ and shRNA#2 5′-AAATTCATCAGAGATAGTCAA-3′ [57], and human TLR3 shRNA#1 5′-GGTATAGCCAGCTAACTAG-3′ and shRNA#2 5′-ACTTAAATGTGGTTGGTAA-3′ and human MDA5 shRNA#1 5′-CCAACAAAGAAGCAGTGTATA-3′ [58] and luciferase (Luc) control shRNA 5′-CTTACGCTGAGTACTTCGA-3′. A549 cell lines stably expressing STAT1-WT, STAT1-2C, SOCS-1 or empty vector (EV) were generated by infecting the cells with retroviruses encoding these genes in pMIG vector as previously described [13].

### Mouse experiments

Female BALB/c mice (5–6 weeks old, 18–20 g) were provided by Vital River Laboratory Animal Center (Beijing, China). To determine the 50% mouse lethal dose (MLD50) of the virus, six groups of five mice were inoculated intranasally with 10-fold serial dilutions of virus. MLD50 titres were calculated by the method of Reed and Muench [59]. For infection, mice were inoculated intranasally with 1×10^5^ plaque-forming units (pfu) of the A/WSN/33 virus. For the peptide treatment, 2 days before viral infection, mice were pre-administrated intraperitoneally (i.p.) once a day with peptide using 5 mg/kg body weight. On the indicated day of post-infection (p.i.), the mice were then euthanized and their lungs were removed for further analysis by Western blotting and RT-PCR.

### Generation of SOCS-1-knockdown transgenic mice

SOCS-1-knockdown transgenic mice were generated by the microinjection method as previously described [35], [36]. Briefly, shRNA-expressing vector targeting mouse SOCS-1 was linearized by *Sca* I. The transgenic mice were generated by the microinjection and genotyped by PCR using specific primers of up-stream: 5′-AAATCCTGGTTGCTGTCTCTTTATGG-3′ and down-stream: 5′-GGAAGGTCCGCTGGATTGA-3′. A 350 bp fragment of the shRNA cassette was amplified, which represented integration of the transgenic DNA. Transgenic mice were analyzed by Western blotting using the anti-SOCS-1 antibody. The transgenic founders with high interference efficiency were selected and maintained on a BALB/c genetic background.

### Statistical analysis

Comparison between groups was made using Student's t-test. Data represent the mean ± SD. Differences were considered statistically significant with P<0.05.

## Supporting Information

Figure S1 **IAV infection induces robust expression of IFN-λs in A549 cells mainly through a RIG-I-dependent pathway.** (**A**) The differentially expressed genes in A549 cells infected with or without A/WSN/33 influenza virus were analyzed by cDNA microarray in our previous study (ncbi.nlm.nih.gov; access number GSE32878). Shown are representative genes whose expressions were most significantly changed. (**B, C**) A549 cells were infected with or without WSN virus (MOI = 1) for 15 h, the expression of IL-28A/B and IL-29 was examined by RT-PCR (B) and IL-29 in supernatants was measured by ELISA (C). (**D**) A549 cells were transfected with indicated amount of “Viral RNA” using Lipofectamine 2000 (L2000). After 4 h post transfection, ELISA was performed to examine the expression of IL-29. (**E**) A549 cells were transfected with indicated amount of WSN genomic RNA (VG-RNA) as described in D. The expression of IL-29 and Mx1 was examined by RT-PCR. (**F**) ELISA was performed to examine the expression of IL-29 in supernatants from cells treated as described in Figure 1E. (**G**) A549 cells expressing shRNAs targeting MDA5 or luciferase (Luc) were infected with or without the WSN, and then the expression of IL-28A/B and IL-29 was examined by RT-PCR. (**H**) IL-29 levels and RIG-I/TLR3/MDA5 levels of infected cells in (G) and Figure 1G-H were quantitated by densitometry, and normalized to control GAPDH levels as described in Figure 2D. Genes expression levels in luciferase A549 cells were set to 100%. Plotted are the average levels from three independent experiments. The error bars represent the S.E. Statistical significance of change was determined by Student's t-test (*P<0.05, **P<0.01).
(TIF)

Figure S2 **IAV-induced-SOCS-1 mainly regulates the autocrine cytokine signaling.** (**A, B**) A549 cells were infected with WSN (MOI = 1 in (A); MOI = 0.5 in (B)) for 15 hrs or uninfected. Immunofluorescence staining was performed using anti-SOCS1 (mouse antibody) and NP (rabbit antibody) (A) or anti-pSTAT1 (rabbit antibody) and NS1 (mouse antibody) (B) to detect the expression of these proteins in cells. More than 70% of A549 cells were infected when an MOI of 1 pfu per cell was used to infect the cells for 15 hours and increased expression of SOCS-1 occurred specifically in infected cells (A). In addition, levels of phosphor-STAT1 were markedly lower in infected cells than those in non-infected cells (B). The nuclei were stained with DAPI. Bar, 10 µm. (**C**) Supernatants derived from IAV-infected A549 cells (15 h p.i.) were collected and used to stimulate the native A549 cells for indicated time. Cells were lysed and the expression of SOCS-1 was detected by RT-PCR. (**D**) ELISA was performed to examine the expression of IL-29 in A549 cells infected with or without WSN (MOI = 1) for indicated time.
(TIF)

Figure S3 **Forced activation of cytokine signaling slightly reduced expression of type I IFN but increased expression of OAS-2 and Mx1 at early time point post infection.** (**A, B**) A549 cells stably expressing shRNAs targeting luciferase or SOCS-1 (A) and A549 cells stably expressing empty vector (EV), STAT1-WT (WT), or active form of STAT1 (STAT1-2C) (B) were infected with or without WSN (MOI = 1) for 15 h. The mRNA levels of IFN-α and IFN-β were examined by RT-PCR. (**C**) IFN-α and IFN-β levels of infected cells in (A) and (B) were quantitated by densitometry, and normalized to control GAPDH levels as described. Plotted are the average levels from three independent experiments. The error bars represent the S.E. (**D, E**) A549 cell lines described in (A) and (B) were infected with or without WSN (MOI = 1) for 6 h. Then mRNA levels of OAS-2 and Mx1 were examined by RT-PCR. (**F**) Mx1 and OAS-2 levels of infected cells in (D) and (E) were quantitated by densitometry, and normalized to control GAPDH levels as described. Plotted are the average levels from three independent experiments. The error bars represent the S.E. (**G, H**) Forced activation of cytokine signaling had no effects on levels of viral RNA and PRRs. Experiments were carried out as described in (A) and (B). mRNA levels of viral NS1 (G, H) and Pattern-Recognition Receptors (PRRs) including TLR3, RIG-I (H) were examined by RT-PCR.
(TIF)

Figure S4 **Disruption of IFN-λ signaling pathway results in activation of NF-κB during IAV infection.** (**A**) A549 cells over-expressing SOCS-1 (S1) or empty vector (EV) were infected with WSN for 15 h or uninfected. Cell lysates were analyzed by Western blotting using indicated antibodies. (**B**) 293T cells were co-transfected with pNFκB-Luc, pRL-TK and pMIG-SOCS-1 or control empty vector (EV) for 10 hrs. Then cells were uninfected or infected with IAV for 15 h and relative luciferase activity was measured. (**C, D**) Experiments were carried out as described in Figure 6 H and I, the nuclear translocation of p65 was counted under fluorescence microscope. Plotted are the average percentages of cells containing nuclear p65 from three independent experiments. The error bars represent the S.E.
(TIF)

Figure S5 **Inhibition of JAK-STAT by SOCS-1 contributes to IAV-induced IFN-λ overproduction and body weight loss of mice.** (**A, B**) BALB/c mice were infected intranasally with WSN virus (1×10^5^ PFU) for indicated time. Lung of the mice were lysed and RT-PCR (A) or real-time PCR (B) were performed to examine the expression kinetics of SOCS-1 and IL-28A/B. (**C–D**) Experiments were carried out as described in Figure 7D and G. RT-PCR were performed to examine the expression of mouse IL-28A/B. (**E, F**) Experiments were carried out as described in Figure 7D and G. Mean body weight was measured every day post infection. Plotted are the average percentages of the initial body weight from three independent experiments. The error bars represent the S.E.
(TIF)

Figure S6 **Silencing SOCS-1 reduced body weight loss and IAV pathogenesis in transgenic mice.** (**A**) The wild type (WT) mice and SOCS-1-knockdown transgenic mice (TG) were inoculated intranasally with WSN (1×10^5^ PFU). On Day 3 p.i., lungs of WT and TG mice were stained with haematoxylin and eosin (HE) for microscope examination (magnification is 400). (**B, C**) The wild type (WT) mice and SOCS-1-knockdown transgenic mice (TG) were inoculated intranasally with WSN (1×10^5^ PFU). Mean body weight was measured as described in Figure S5E. Plotted are the average percentages of the initial body weight from three independent experiments. The error bars represent the S.E. Survival of wild-type mice and SOCS-1-knockdown transgenic mice were monitored every day (C).
(TIF)
