# Correction: Suppression of Interferon Lambda Signaling by SOCS-1 Results in Their Excessive Production during Influenza Virus Infection

**DOI:** 10.1371/journal.ppat.1005402

**Published:** 2016-01-14

**Authors:** Haitao Wei, Song Wang, Qinghuang Chen, Yuhai Chen, Xiaojuan Chi, Lianfeng Zhang, Shile Huang, George F. Gao, Ji-Long Chen

The authors would like to correct Figs 1, 2, 5 and 8, as errors were inadvertently introduced in the preparation of these figures for publication. In Fig 1G, the IL-29 panel was duplicated to create the IL28A/B panel; the correct IL28A/B panel has been inserted into the corrected Fig 1G. In Fig 2B, the STAT1 panel from Fig 2A was duplicated to create the STAT1 panel in Fig 2B; the correct STAT1 panel has been inserted into the corrected Fig 2B. In Fig 5D, the IL-28A/B panel and the IL-29 panel appeared very similar; Fig 5D has been re-assembled for clarity. In Fig 8F, the mSOCS-1 panel was duplicated to create the IL28A/B panel; the correct IL28A/B panel has been inserted into the corrected Fig 8F. The corrected version of Fig 1, 2, 5 and 8 can be viewed here.

The authors confirm that these changes do not alter their findings. The authors have provided raw, uncropped blots as Supporting Information.

**Fig 1:**
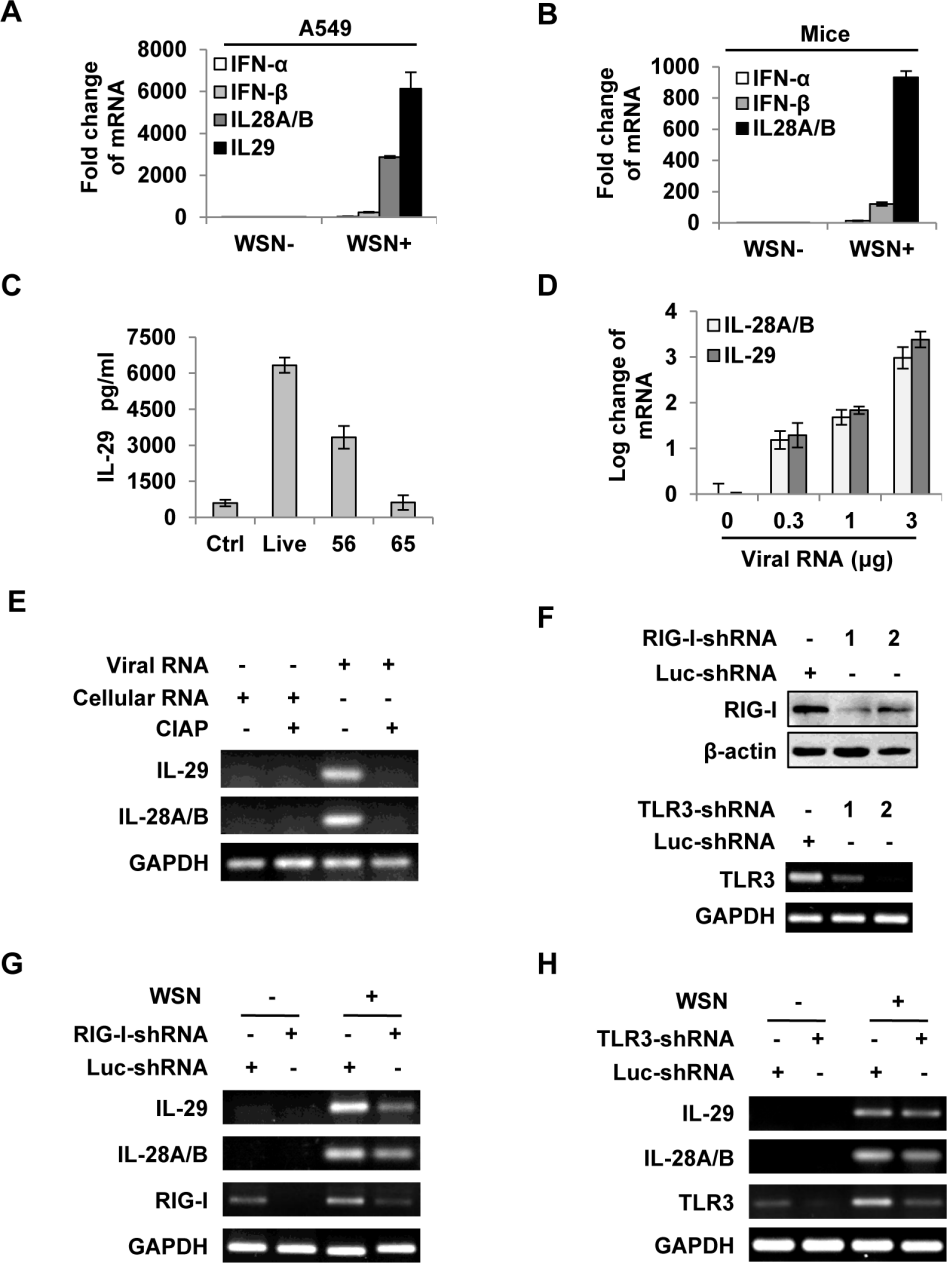
IAV infection induces robust expression of IFN-λ in alveolar epithelial cells mainly through a RIG-I-dependent pathway. **(A)** A549 cells infected with or without WSN virus (MOI = 1) for 15 h, mRNA levels of IFN-α, β and λ were examined by real-time PCR. **(B)** BALB/c mice were infected intranasally with or without WSN virus (1×10^5^ PFU). On day 3 p.i., lungs were lysed, and the mRNA levels of IFN-α, β and λ were examined by real-time PCR. **(C)** A549 cells were uninfected (Ctrl) or infected with WSN that was untreated (Live) or treated at 56°C or 65°C. IL-29 levels in supernatants from A549 cells at 15 h p.i. were measured by ELISA. **(D)** Different amounts of total RNA (“Viral RNA”) from A549 cells infected with the IAV were transfected into native A549 cells using Lipofectamine 2000 (L2000). Expression of IL-28A/B and IL-29 in transfected A549 cells was examined by real-time PCR at 4 h p.i. **(E)** “Viral RNA” and “Cellular RNA” (total RNA from uninfected control A549 cells) treated with or without calf intestine alkaline phosphatase (CIAP) were transfected into native A549 cells. RT-PCR was performed to examine the expression of IL-28A/B and IL-29. **(F)** shRNA based-knockdown of RIG-I and TLR3 were analyzed by Western blotting or RT-PCR to determine the interference efficiency. **(G-H)** A549 cells expressing shRNAs targeting RIG-I (G), TLR3 (H) or luciferase (Luc) were infected with or without WSN, and then the expression of IL-28A/B and IL-29 was examined by RT-PCR. Results are representative of three independent experiments.

**Fig 2:**
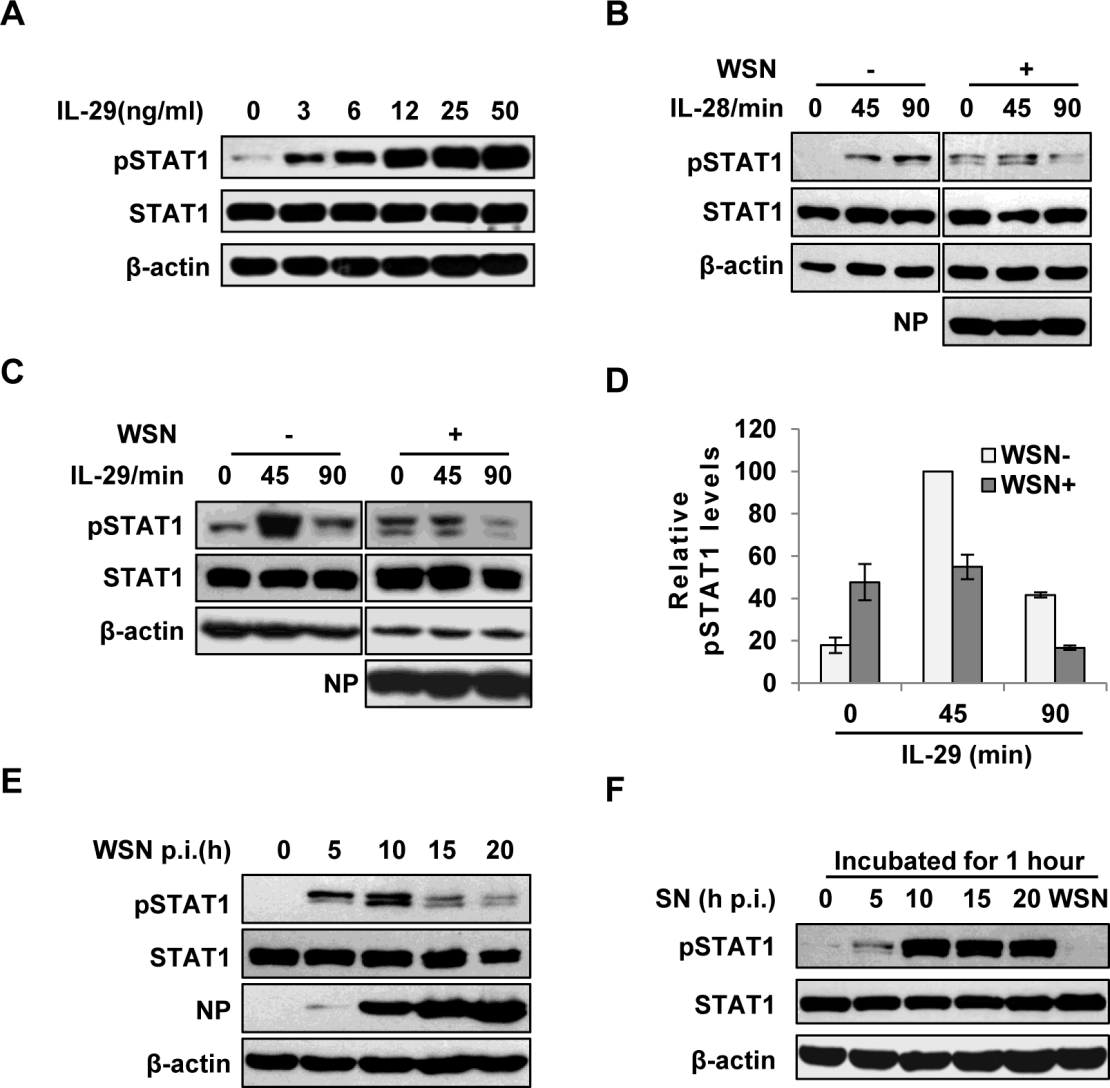
IAV inhibits IL-29-induced STAT1 phosphorylation in A549 cells. **(A)** A549 cells were treated with IL-29 at final concentration of 3, 6, 12, 25, and 50 ng/ml for 45 min, followed by immunoblotting with indicated antibodies. **(B, C)** A549 cells infected with WSN (MOI = 1) for 15 h (WSN+) or non-infected (WSN-) were stimulated with human IL-28A (B) or IL-29 (50 ng/ml) (C) for indicated time. Cell lysates were analyzed by Western blotting using indicated antibodies. **(D)** Levels of phosphorylated STAT1 in (C) were quantitated by densitometry, and normalized to STAT1 expression and control β-actin levels. In each experiment, the highest level of STAT1 phosphorylation is 100. Plotted are the average levels from three independent experiments. The error bars represent the S.E. **(E)** A549 cells were infected with WSN (MOI = 1), lysed at the 0, 5, 10, 15 and 20 h p.i., and analyzed by Western blotting using indicated antibodies. **(F)** A549 cells were either stimulated by supernatant (SN) culture medium from IAV-infected cells in (E) or infected with WSN for 1 h, followed by Western blotting with indicated antibodies.

**Fig 5:**
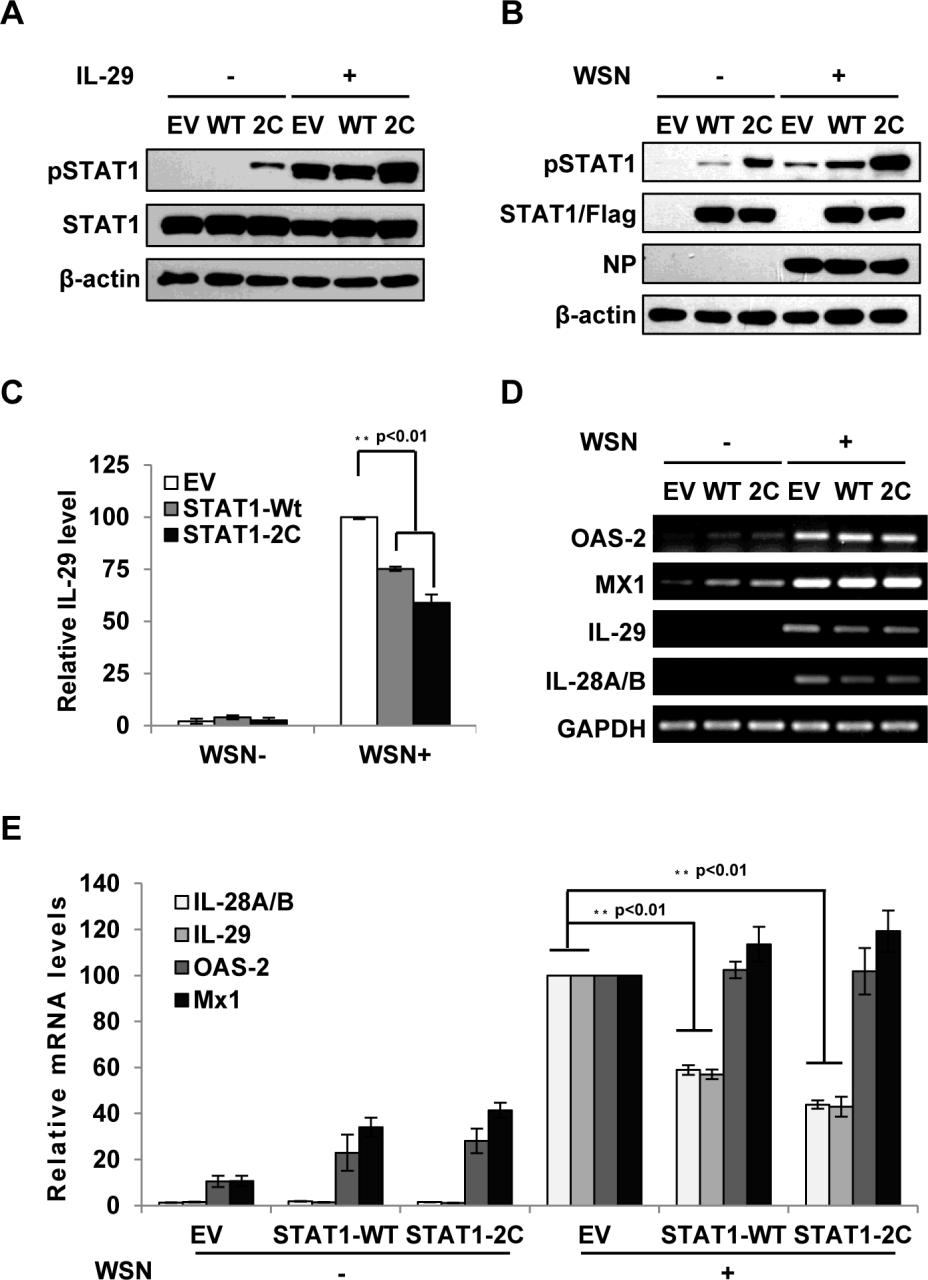
Forced activation of STAT1 causes a significant decrease in IFN-λ expression during IAV infection. **(A)** A549 cell lines stably expressing STAT1-WT, STAT1-2C or empty vector (EV) were treated with or without IL-29 (50 ng/ml) for 45 min. Cell lysates were analyzed by Western blot using indicated antibodies. **(B-D)** A549 cell lines described in (A) were infected with or without WSN virus for 15 h. Subsequently, the cell lysates were analyzed by Western blot probed with indicated antibodies (B), and the protein levels of IL-29 in the cell culture supernatants were examined by ELISA (C). In panel C, IL-29 levels produced by infected cells expressing EV were set to 100%. Plotted are the average results from three independent experiments. The error bars represent the S.E. mRNA levels of OAS-2, Mx1, IL-28A/B and IL-29 were measured by RT-PCR (D). **(E)** IFN-λ levels and OAS-2 and Mx1 levels in (D) were quantitated by densitometry, and normalized to GAPDH levels as described in Fig 2D. Plotted are the average levels from three independent experiments. The error bars represent the S.E. Statistical significance of change was determined by Student’s t-test (*P<0.05, **P<0.01).

**Fig 8:**
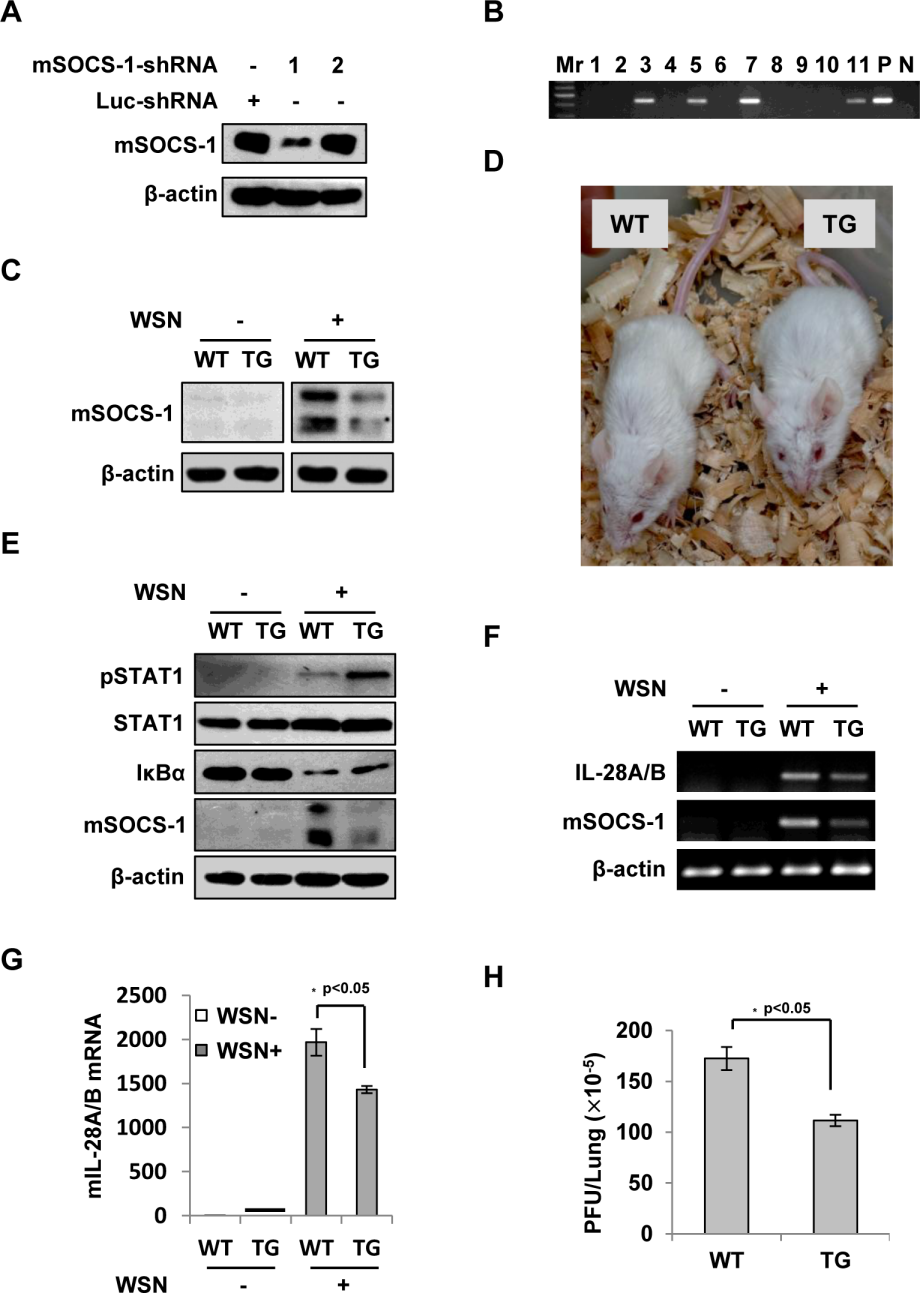
Silencing SOCS-1 causes a significant reduction of IFN-λ expression in transgenic mice during IAV infection. **(A)** Immunoblotting was performed to test shRNA-based knockdown of mouse SOCS-1 in transfected cell line. **(B)** The SOCS-1-knockdown transgenic mice were genotyped by PCR. Shown is representative genotyping of SOCS-1-knockdown transgenic mice. Numbers 1–11, representative transgenic mice and wild type littermates; P, positive control; N, negative control. **(C)** SOCS-1 expression in representative tissues (lung) from SOCS-1-knockdown transgenic mice (TG) and wild-type littermates (WT) was examined by immunoblotting after WSN infection. **(D)** The transgenic founders with high interference efficiency were selected and maintained on a BALB/c genetic background. Shown is a representative photograph of SOCS-1-knockdown transgenic mouse and wild-type littermate. **(E-H)** WT and TG mice were infected with or without WSN virus as described in Fig 7. On Day 3 p.i., lungs were lysed and analyzed by Western blotting with indicated antibodies (E), IL-28A/B expression was examined by RT-PCR (F) and real-time PCR (G), and viral titers in lungs of WT and TG mice were examined by plaque assay and values are shown as mean ± SD (H).

## Supporting Information

S1 Fig Raw data for Fig 1G. A549 cells expressing shRNAs targeting RIG-I or luciferase (Luc) were infected with or without WSN virus, and then the expression of IL-28A/B, RIG-I and IL-29 was determined by RT-PCR and examined by agarose gel electrophoresis as indicated.
(PPT)

S2 Fig Raw data for Fig 2B. A549 cells infected with WSN virus (MOI = 1) for 15 h or non-infected were stimulated with human IL-28A for indicated time as described in Fig 2B. Cell lysates were analyzed by Western blotting using anti-β-actin, anti-phosphorylated STAT1 (Tyr701), anti-STAT1 and anti-viral NP antibodies.
(PPTX)

S3 Fig Raw data for Fig 5D. A549 cell lines stably expressing STAT1-WT, STAT1-2C or empty vector (EV) were infected with or without WSN virus for 15 h. mRNA levels of IL-28A/B, GAPDH and IL-29 were measured by RT-PCR and examined by agarose gel electrophoresis.
(PPT)

S4 Fig Raw data for Fig 8F. WT and TG mice were infected with or without WSN virus intranasally (1×10^5^ PFU). On Day 3 p.i., lungs were lysed and expression of IL-28A/B, mSOCS1 and β-actin was examined by RT-PCR followed by agarose gel electrophoresis.
(PPT)
