# Supplementary figures and images for: Suppression of Interferon Lambda Signaling by SOCS-1 Results in Their Excessive Production during Influenza Virus Infection
Source: PLoS Pathog. 2014 Jan 2;10(1):e1003845. doi: 10.1371/journal.ppat.1003845 (PMC3879354; doi:10.1371/journal.ppat.1003845)

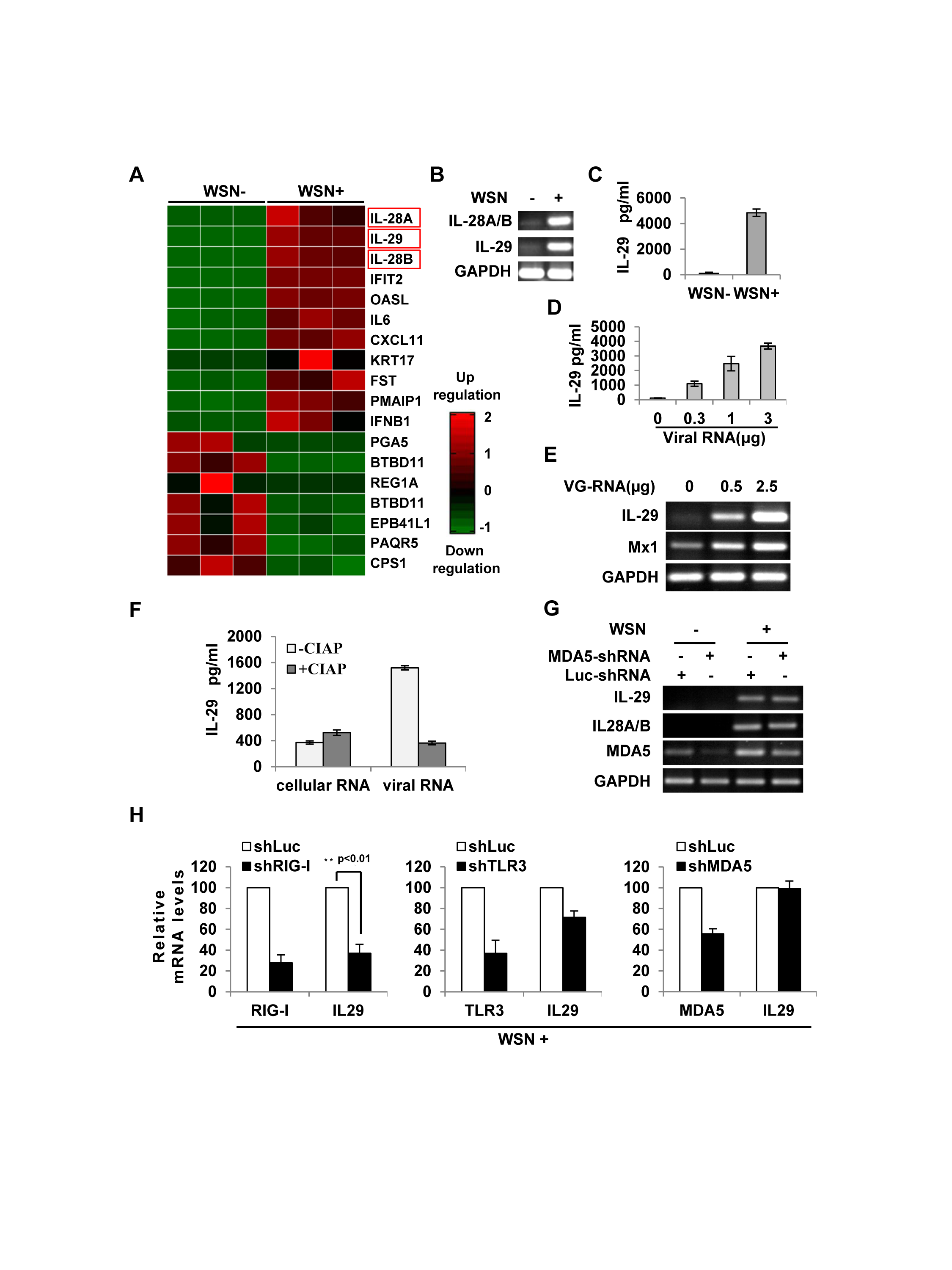

Supplement: Figure S1 — IAV infection induces robust expression of IFN-λs in A549 cells mainly through a RIG-I-dependent pathway. (A) The differentially expressed genes in A549 cells infected with or without A/WSN/33 influenza virus were analyzed by cDNA microarray in our previous study (ncbi.nlm.nih.gov; access number GSE32878). Shown are representative genes whose expressions were most significantly changed. (B, C) A549 cells were infected with or without WSN virus (MOI = 1) for 15 h, the expression of IL-28A/B and IL-29 was examined by RT-PCR (B) and IL-29 in supernatants was measured by ELISA (C). (D) A549 cells were transfected with indicated amount of “Viral RNA” using Lipofectamine 2000 (L2000). After 4 h post transfection, ELISA was performed to examine the expression of IL-29. (E) A549 cells were transfected with indicated amount of WSN genomic RNA (VG-RNA) as described in D. The expression of IL-29 and Mx1 was examined by RT-PCR. (F) ELISA was performed to examine the expression of IL-29 in supernatants from cells treated as described in Figure 1E. (G) A549 cells expressing shRNAs targeting MDA5 or luciferase (Luc) were infected with or without the WSN, and then the expression of IL-28A/B and IL-29 was examined by RT-PCR. (H) IL-29 levels and RIG-I/TLR3/MDA5 levels of infected cells in (G) and Figure 1G-H were quantitated by densitometry, and normalized to control GAPDH levels as described in Figure 2D. Genes expression levels in luciferase A549 cells were set to 100%. Plotted are the average levels from three independent experiments. The error bars represent the S.E. Statistical significance of change was determined by Student's t-test (*P<0.05, **P<0.01). (TIF) [file ppat.1003845.s001.tif]

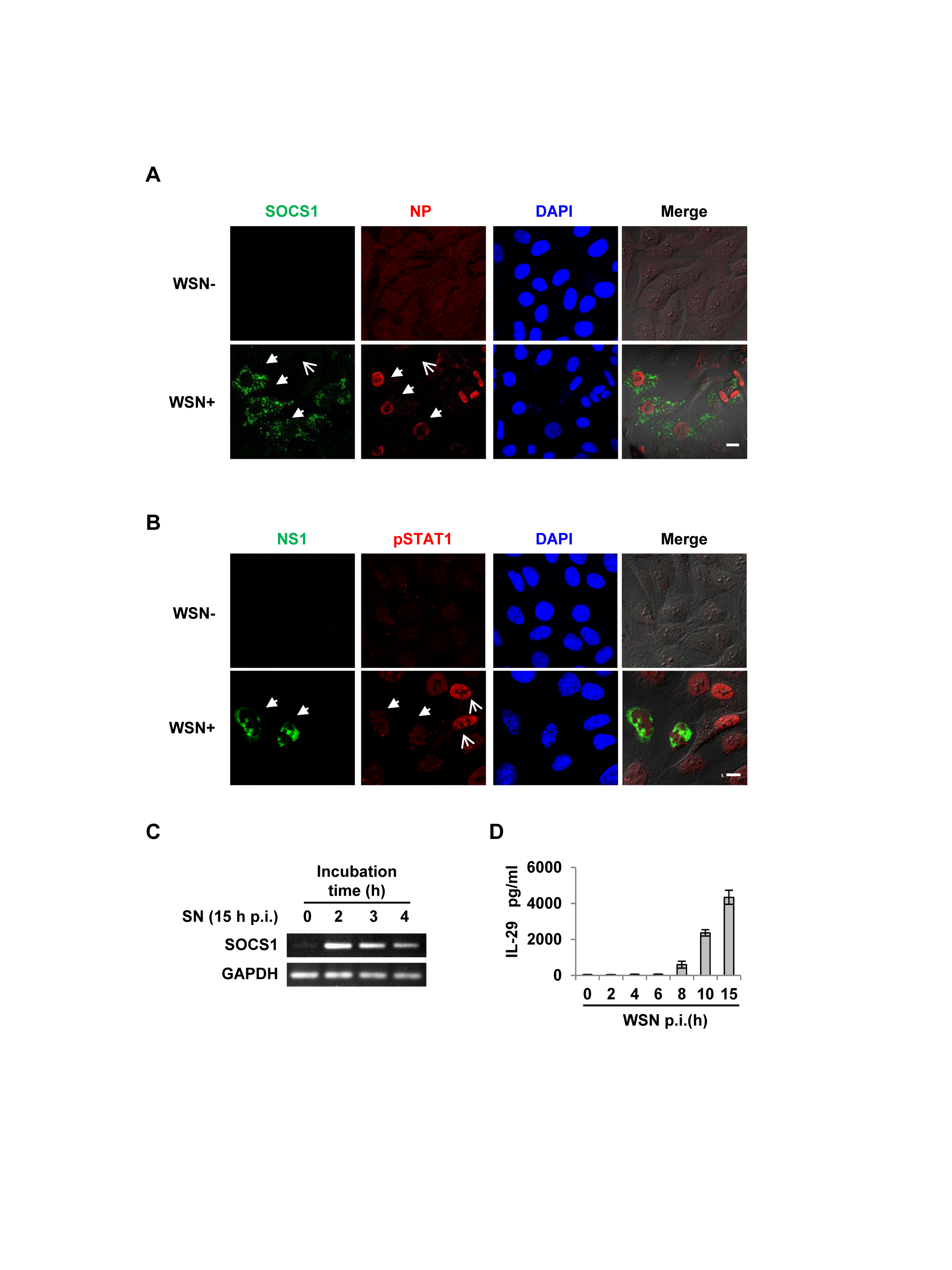

Supplement: Figure S2 — IAV-induced-SOCS-1 mainly regulates the autocrine cytokine signaling. (A, B) A549 cells were infected with WSN (MOI = 1 in (A); MOI = 0.5 in (B)) for 15 hrs or uninfected. Immunofluorescence staining was performed using anti-SOCS1 (mouse antibody) and NP (rabbit antibody) (A) or anti-pSTAT1 (rabbit antibody) and NS1 (mouse antibody) (B) to detect the expression of these proteins in cells. More than 70% of A549 cells were infected when an MOI of 1 pfu per cell was used to infect the cells for 15 hours and increased expression of SOCS-1 occurred specifically in infected cells (A). In addition, levels of phosphor-STAT1 were markedly lower in infected cells than those in non-infected cells (B). The nuclei were stained with DAPI. Bar, 10 µm. (C) Supernatants derived from IAV-infected A549 cells (15 h p.i.) were collected and used to stimulate the native A549 cells for indicated time. Cells were lysed and the expression of SOCS-1 was detected by RT-PCR. (D) ELISA was performed to examine the expression of IL-29 in A549 cells infected with or without WSN (MOI = 1) for indicated time. (TIF) [file ppat.1003845.s002.tif]

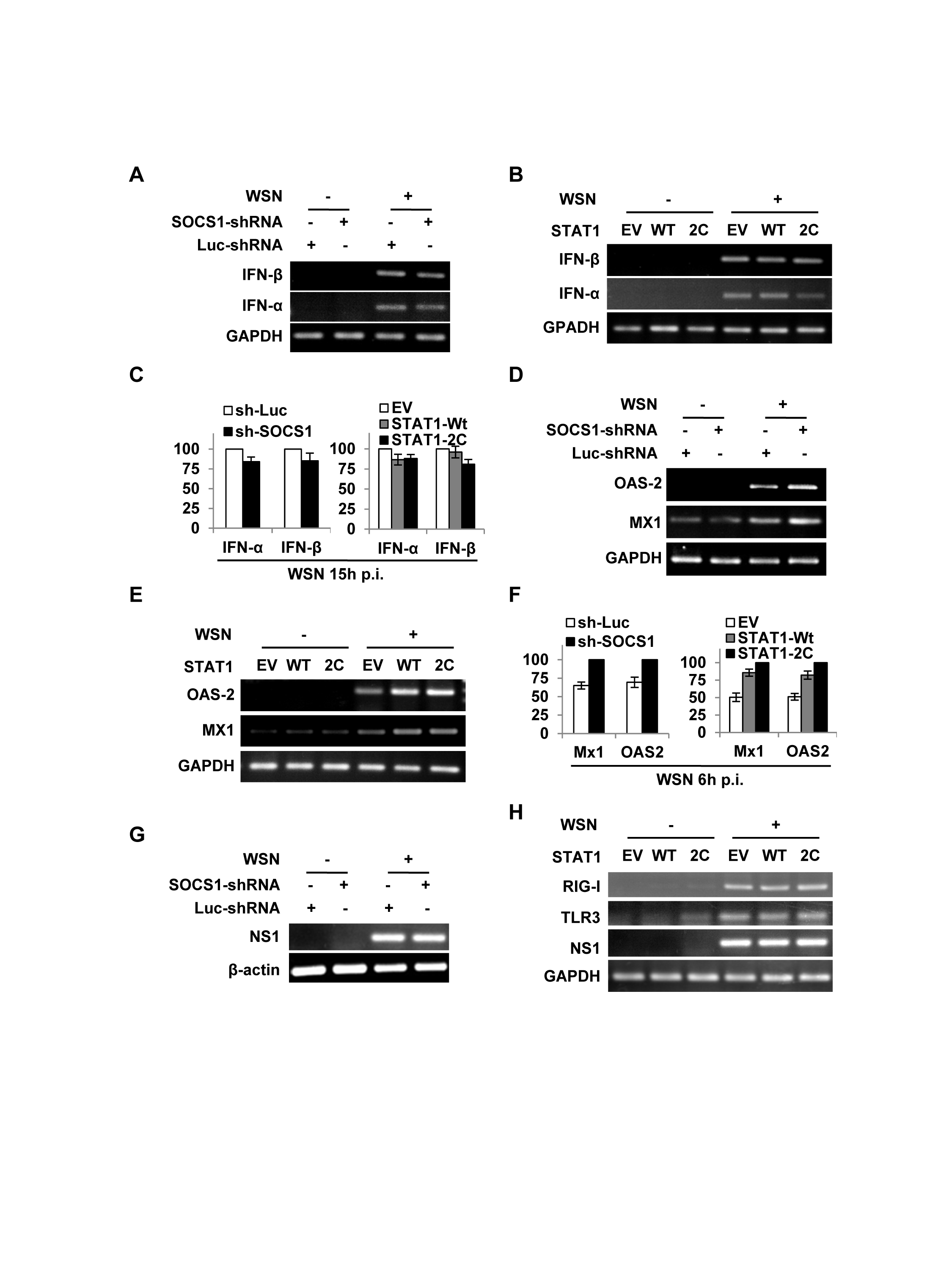

Supplement: Figure S3 — Forced activation of cytokine signaling slightly reduced expression of type I IFN but increased expression of OAS-2 and Mx1 at early time point post infection. (A, B) A549 cells stably expressing shRNAs targeting luciferase or SOCS-1 (A) and A549 cells stably expressing empty vector (EV), STAT1-WT (WT), or active form of STAT1 (STAT1-2C) (B) were infected with or without WSN (MOI = 1) for 15 h. The mRNA levels of IFN-α and IFN-β were examined by RT-PCR. (C) IFN-α and IFN-β levels of infected cells in (A) and (B) were quantitated by densitometry, and normalized to control GAPDH levels as described. Plotted are the average levels from three independent experiments. The error bars represent the S.E. (D, E) A549 cell lines described in (A) and (B) were infected with or without WSN (MOI = 1) for 6 h. Then mRNA levels of OAS-2 and Mx1 were examined by RT-PCR. (F) Mx1 and OAS-2 levels of infected cells in (D) and (E) were quantitated by densitometry, and normalized to control GAPDH levels as described. Plotted are the average levels from three independent experiments. The error bars represent the S.E. (G, H) Forced activation of cytokine signaling had no effects on levels of viral RNA and PRRs. Experiments were carried out as described in (A) and (B). mRNA levels of viral NS1 (G, H) and Pattern-Recognition Receptors (PRRs) including TLR3, RIG-I (H) were examined by RT-PCR. (TIF) [file ppat.1003845.s003.tif]

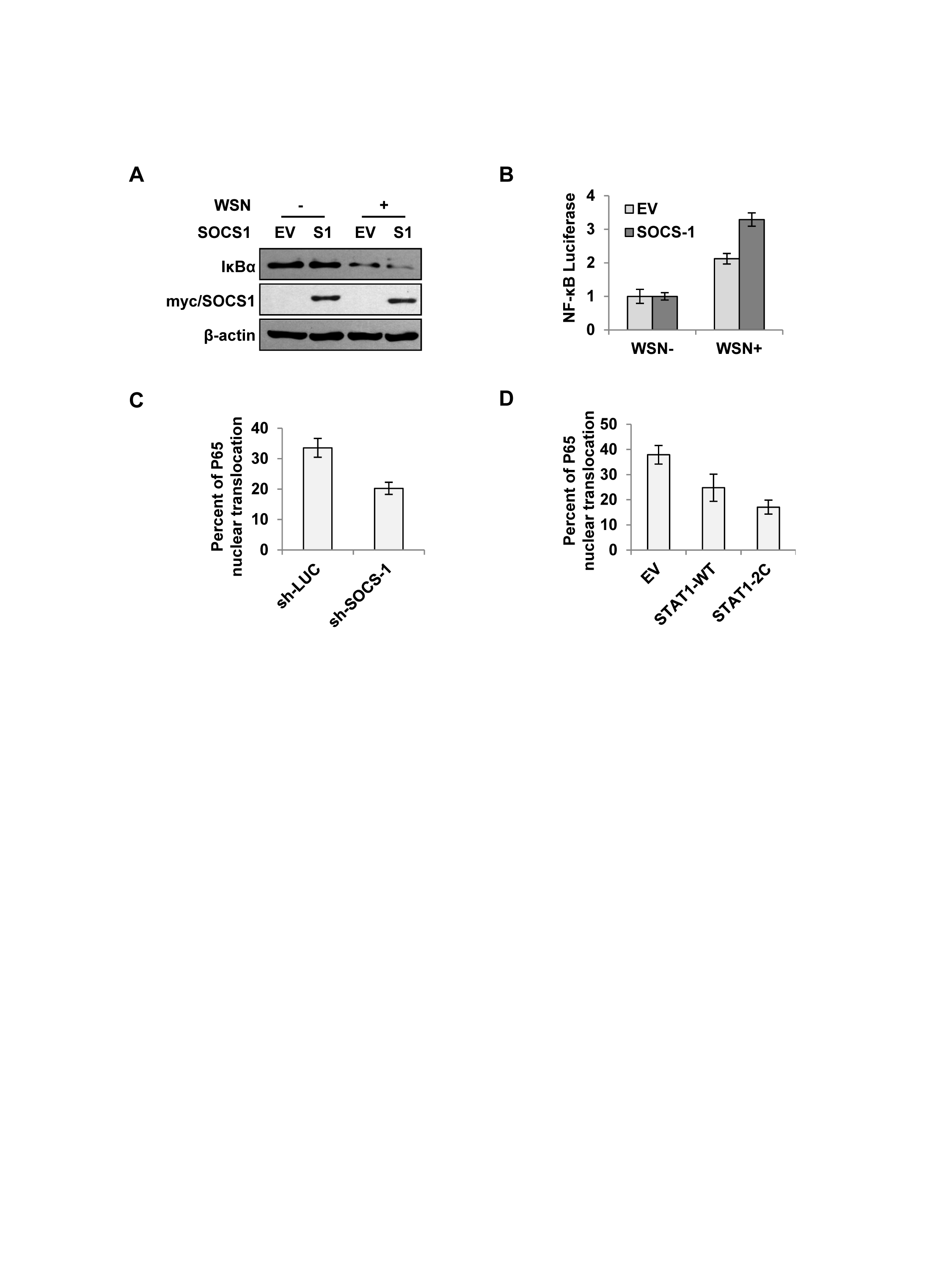

Supplement: Figure S4 — Disruption of IFN-λ signaling pathway results in activation of NF-κB during IAV infection. (A) A549 cells over-expressing SOCS-1 (S1) or empty vector (EV) were infected with WSN for 15 h or uninfected. Cell lysates were analyzed by Western blotting using indicated antibodies. (B) 293T cells were co-transfected with pNFκB-Luc, pRL-TK and pMIG-SOCS-1 or control empty vector (EV) for 10 hrs. Then cells were uninfected or infected with IAV for 15 h and relative luciferase activity was measured. (C, D) Experiments were carried out as described in Figure 6 H and I, the nuclear translocation of p65 was counted under fluorescence microscope. Plotted are the average percentages of cells containing nuclear p65 from three independent experiments. The error bars represent the S.E. (TIF) [file ppat.1003845.s004.tif]

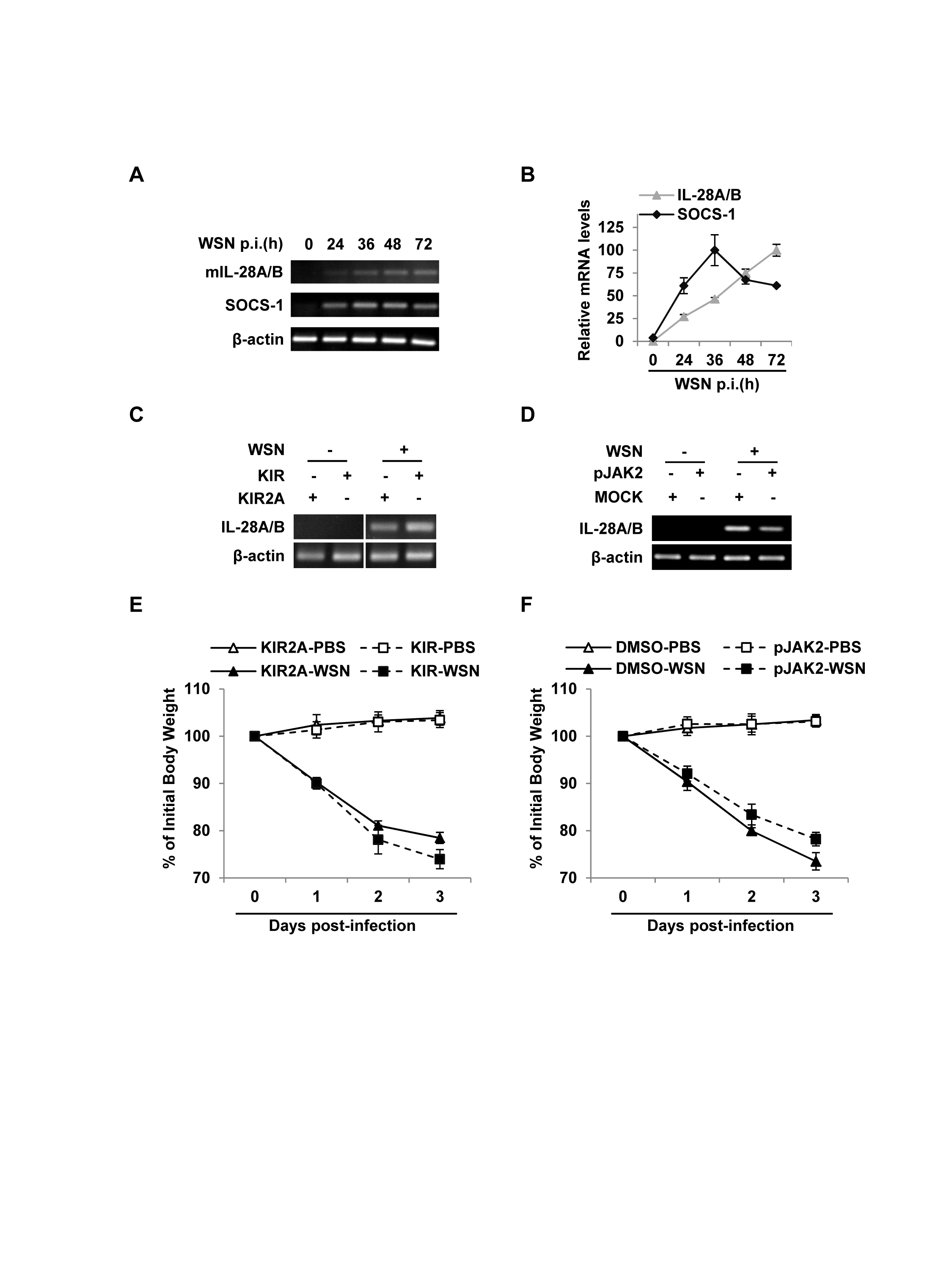

Supplement: Figure S5 — Inhibition of JAK-STAT by SOCS-1 contributes to IAV-induced IFN-λ overproduction and body weight loss of mice. (A, B) BALB/c mice were infected intranasally with WSN virus (1×10^5^ PFU) for indicated time. Lung of the mice were lysed and RT-PCR (A) or real-time PCR (B) were performed to examine the expression kinetics of SOCS-1 and IL-28A/B. (C–D) Experiments were carried out as described in Figure 7D and G. RT-PCR were performed to examine the expression of mouse IL-28A/B. (E, F) Experiments were carried out as described in Figure 7D and G. Mean body weight was measured every day post infection. Plotted are the average percentages of the initial body weight from three independent experiments. The error bars represent the S.E. (TIF) [file ppat.1003845.s005.tif]

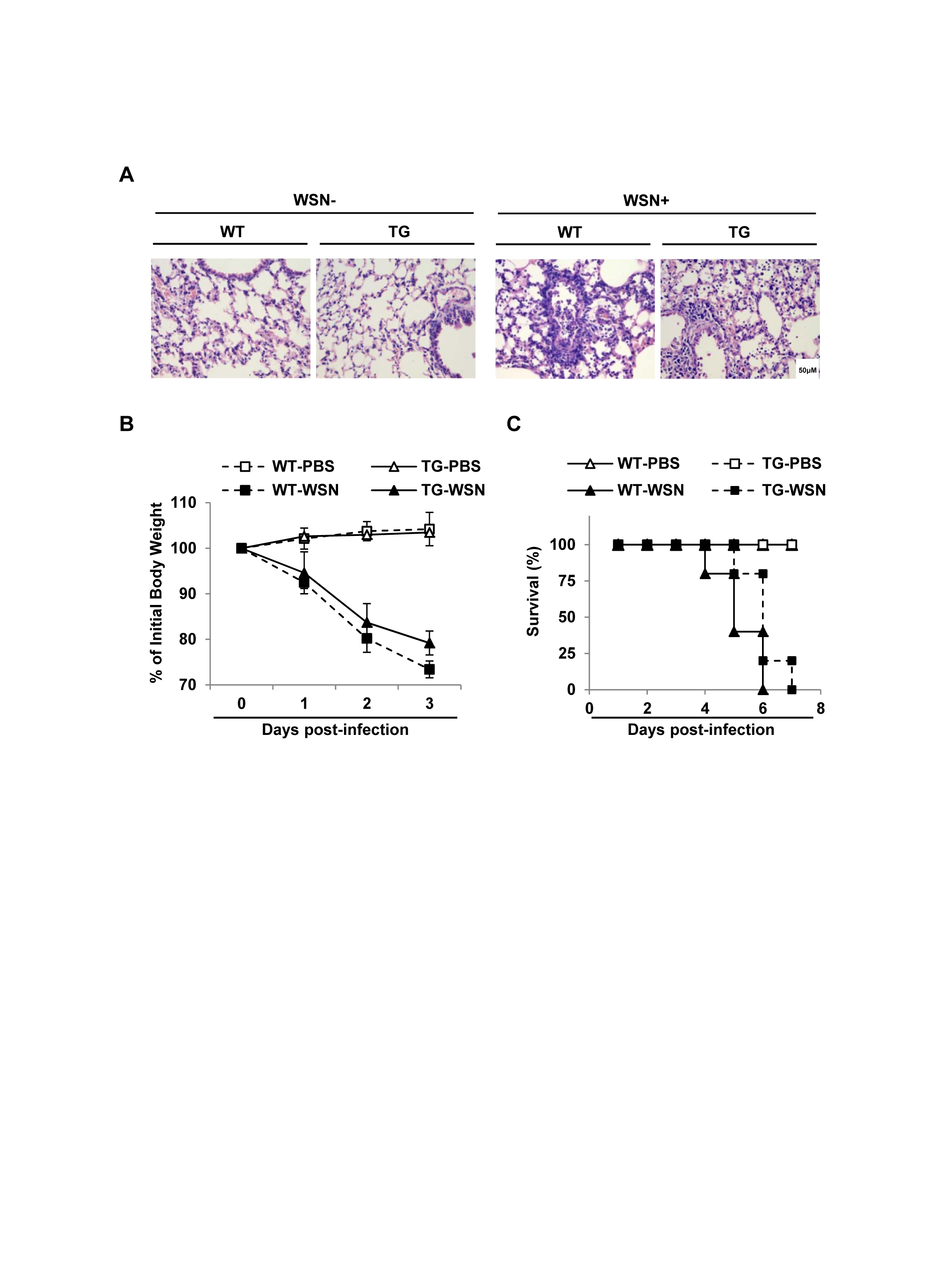

Supplement: Figure S6 — Silencing SOCS-1 reduced body weight loss and IAV pathogenesis in transgenic mice. (A) The wild type (WT) mice and SOCS-1-knockdown transgenic mice (TG) were inoculated intranasally with WSN (1×10^5^ PFU). On Day 3 p.i., lungs of WT and TG mice were stained with haematoxylin and eosin (HE) for microscope examination (magnification is 400). (B, C) The wild type (WT) mice and SOCS-1-knockdown transgenic mice (TG) were inoculated intranasally with WSN (1×10^5^ PFU). Mean body weight was measured as described in Figure S5E. Plotted are the average percentages of the initial body weight from three independent experiments. The error bars represent the S.E. Survival of wild-type mice and SOCS-1-knockdown transgenic mice were monitored every day (C). (TIF) [file ppat.1003845.s006.tif]
