# Supplementary figures and images for: Correction: Suppression of Interferon Lambda Signaling by SOCS-1 Results in Their Excessive Production during Influenza Virus Infection
Source: PLoS Pathog. 2016 Jan 14;12(1):e1005402. doi: 10.1371/journal.ppat.1005402 (PMC4713154; doi:10.1371/journal.ppat.1005402)

## Slide 1
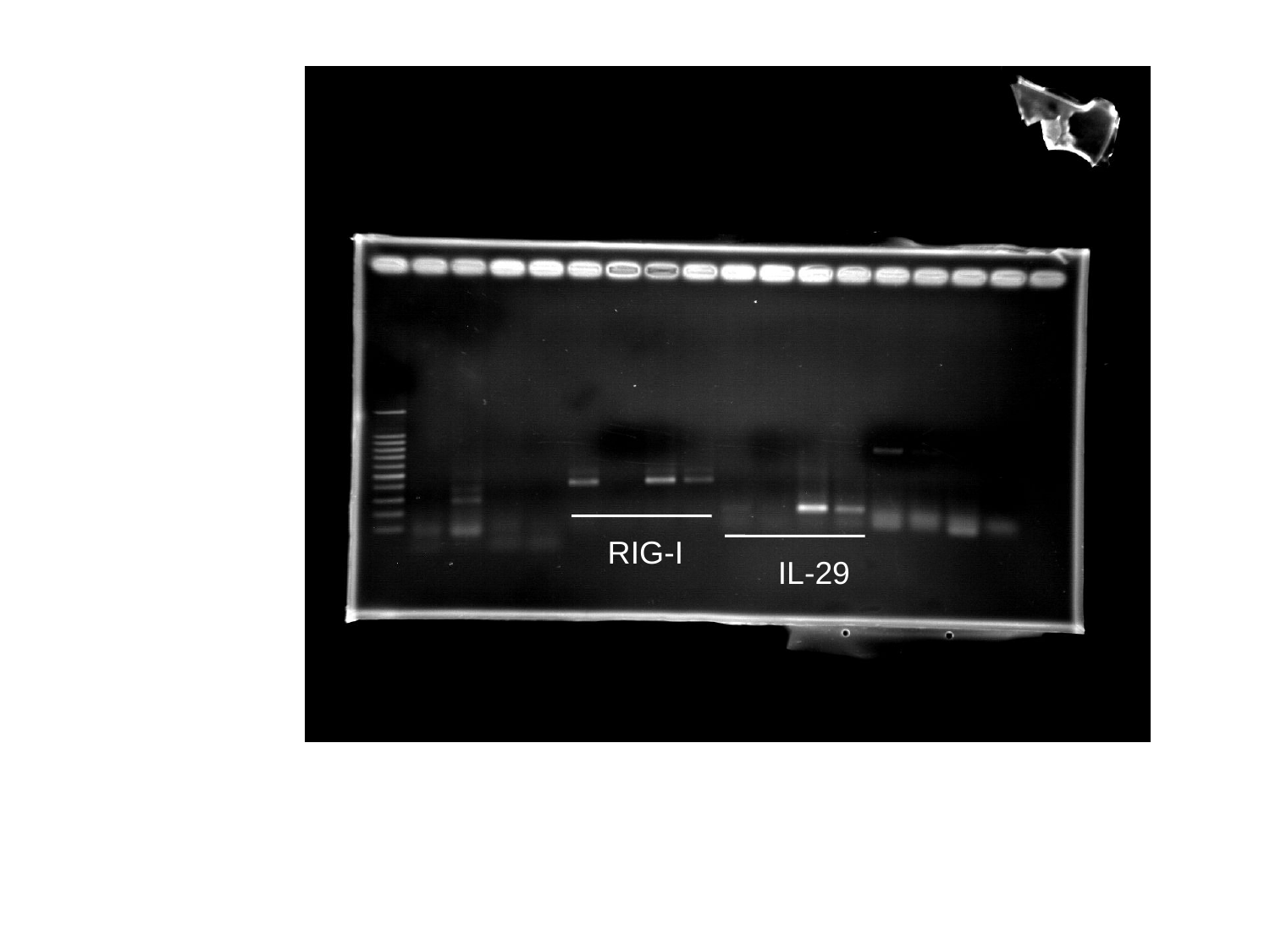

RIG-I
IL-29

## Slide 2
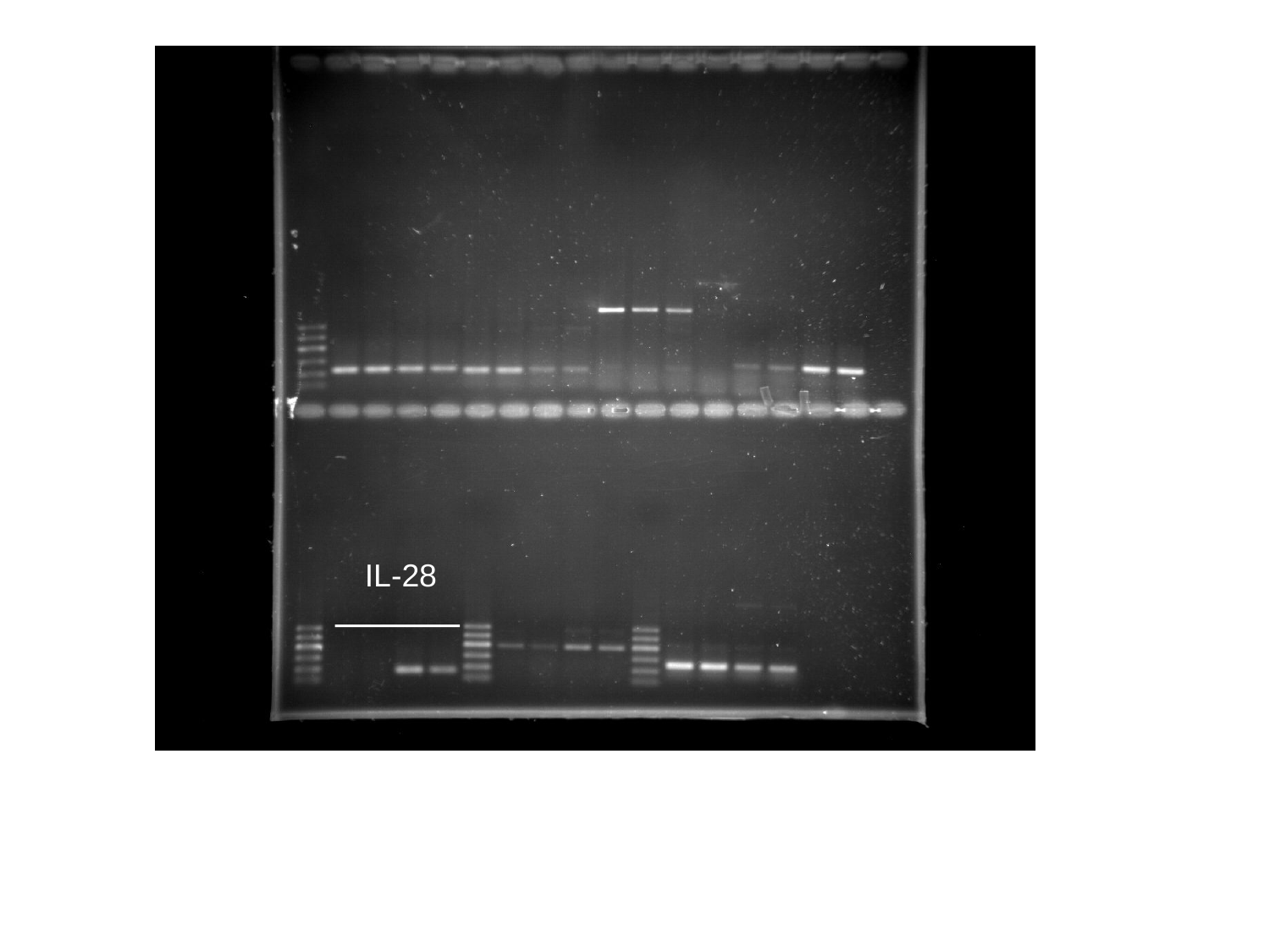

IL-28

Supplement: S1 Fig — A549 cells expressing shRNAs targeting RIG-I or luciferase (Luc) were infected with or without WSN virus, and then the expression of IL-28A/B, RIG-I and IL-29 was determined by RT-PCR and examined by agarose gel electrophoresis as indicated. (PPT) [file ppat.1005402.s001.ppt]

## Slide 1
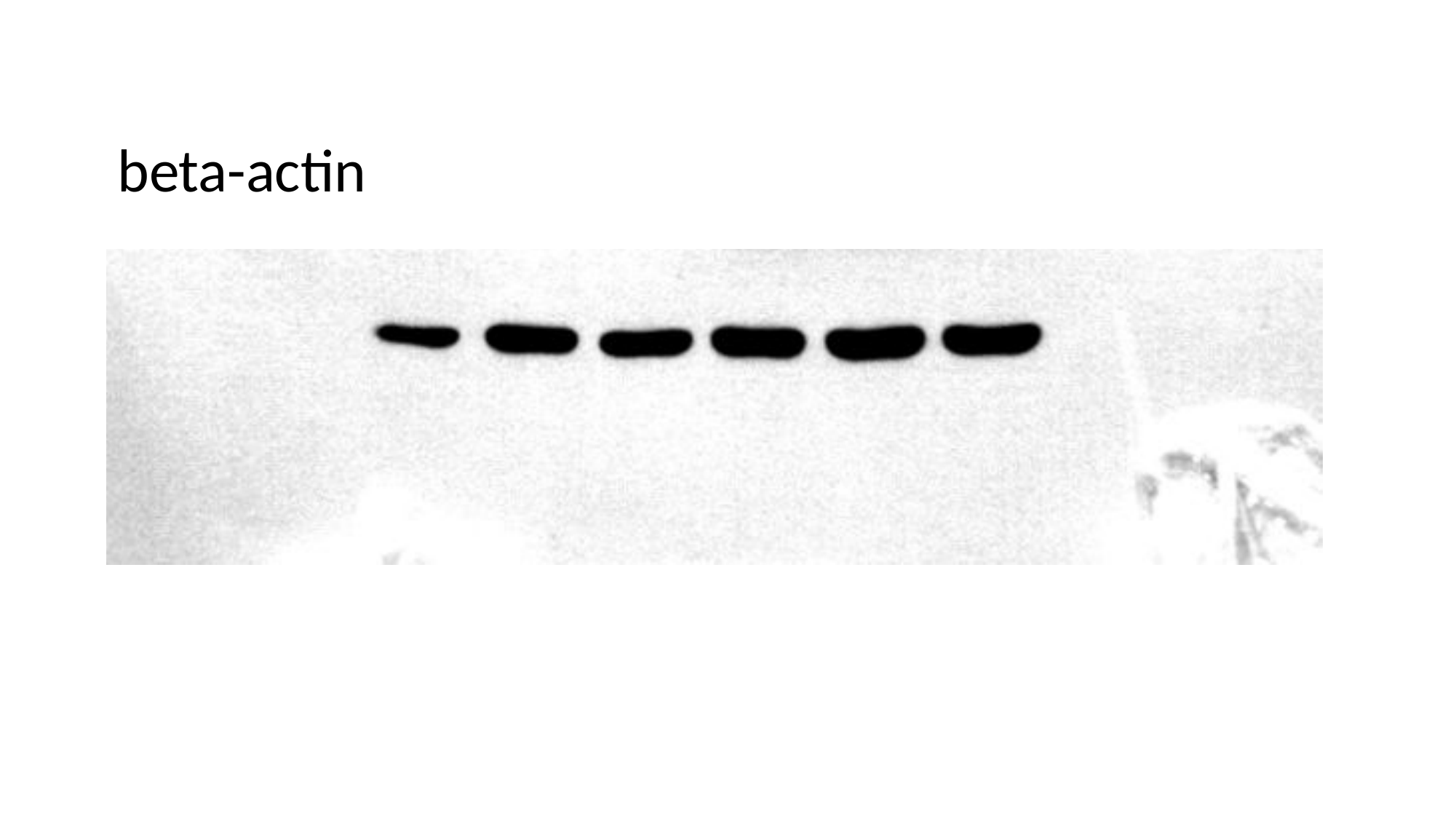

beta-actin

## Slide 2
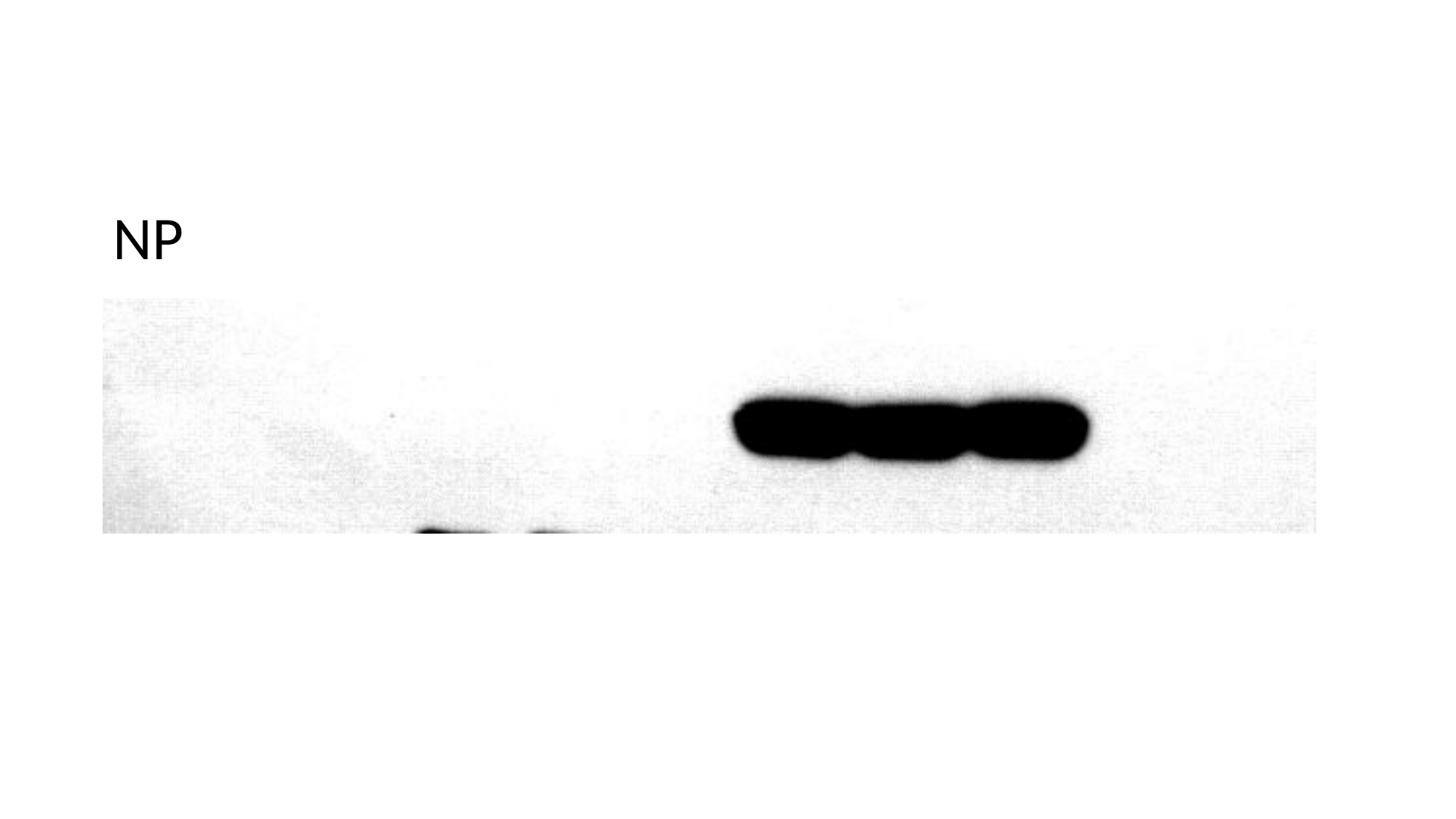

NP

## Slide 3
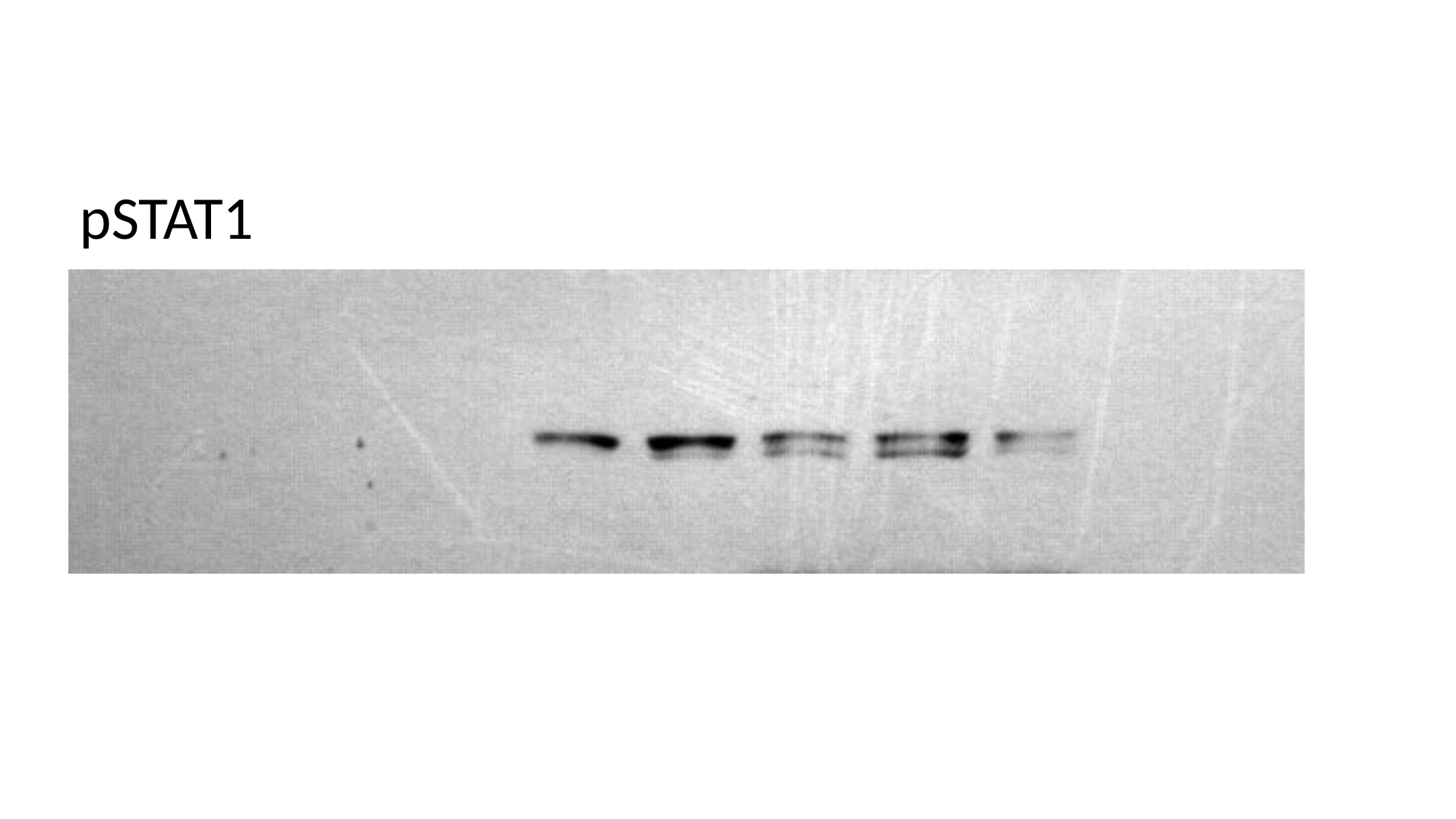

pSTAT1

## Slide 4
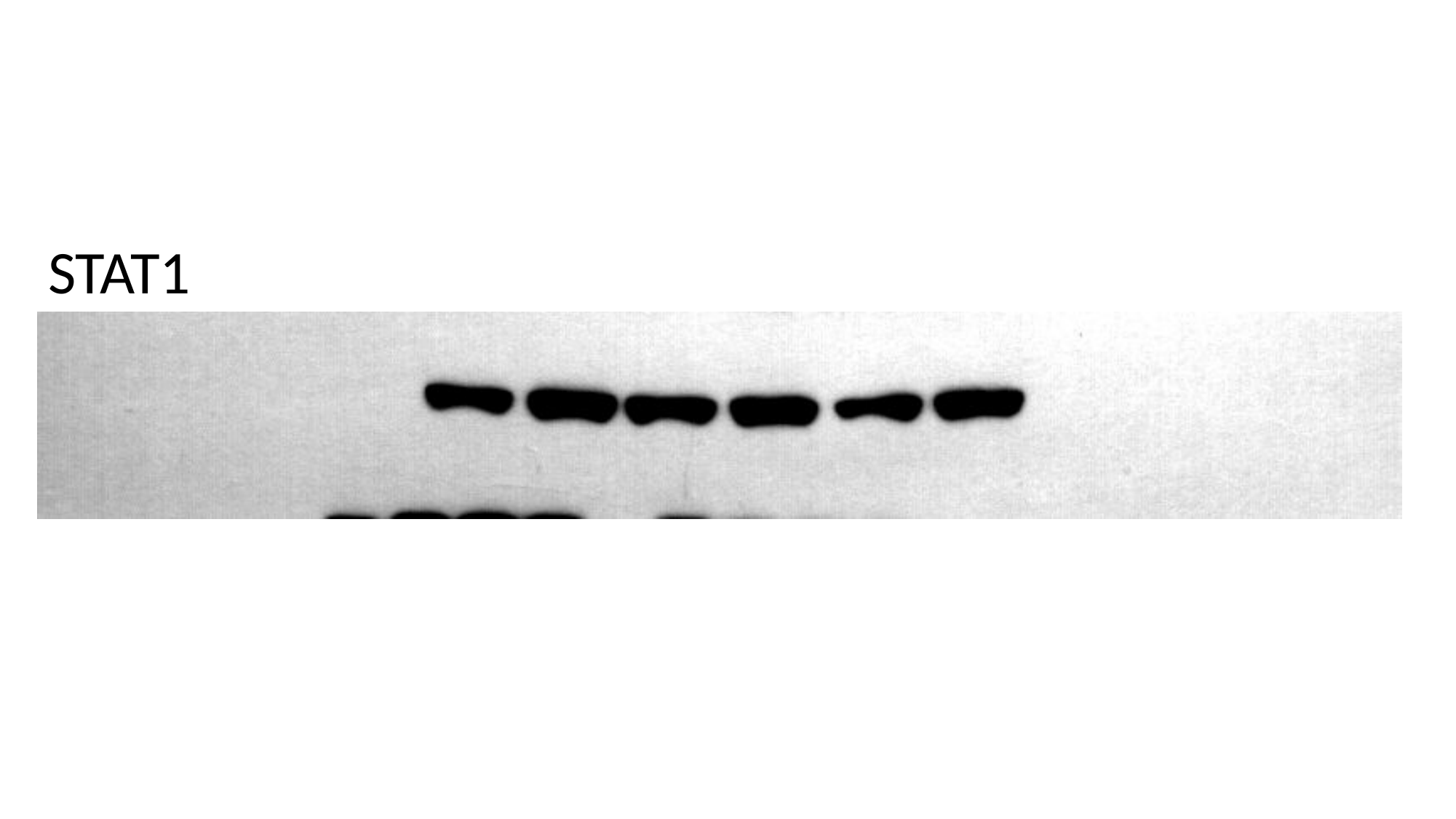

STAT1

Supplement: S2 Fig — A549 cells infected with WSN virus (MOI = 1) for 15 h or non-infected were stimulated with human IL-28A for indicated time as described in Fig 2B. Cell lysates were analyzed by Western blotting using anti-β-actin, anti-phosphorylated STAT1 (Tyr701), anti-STAT1 and anti-viral NP antibodies. (PPTX) [file ppat.1005402.s002.pptx]

## Slide 1
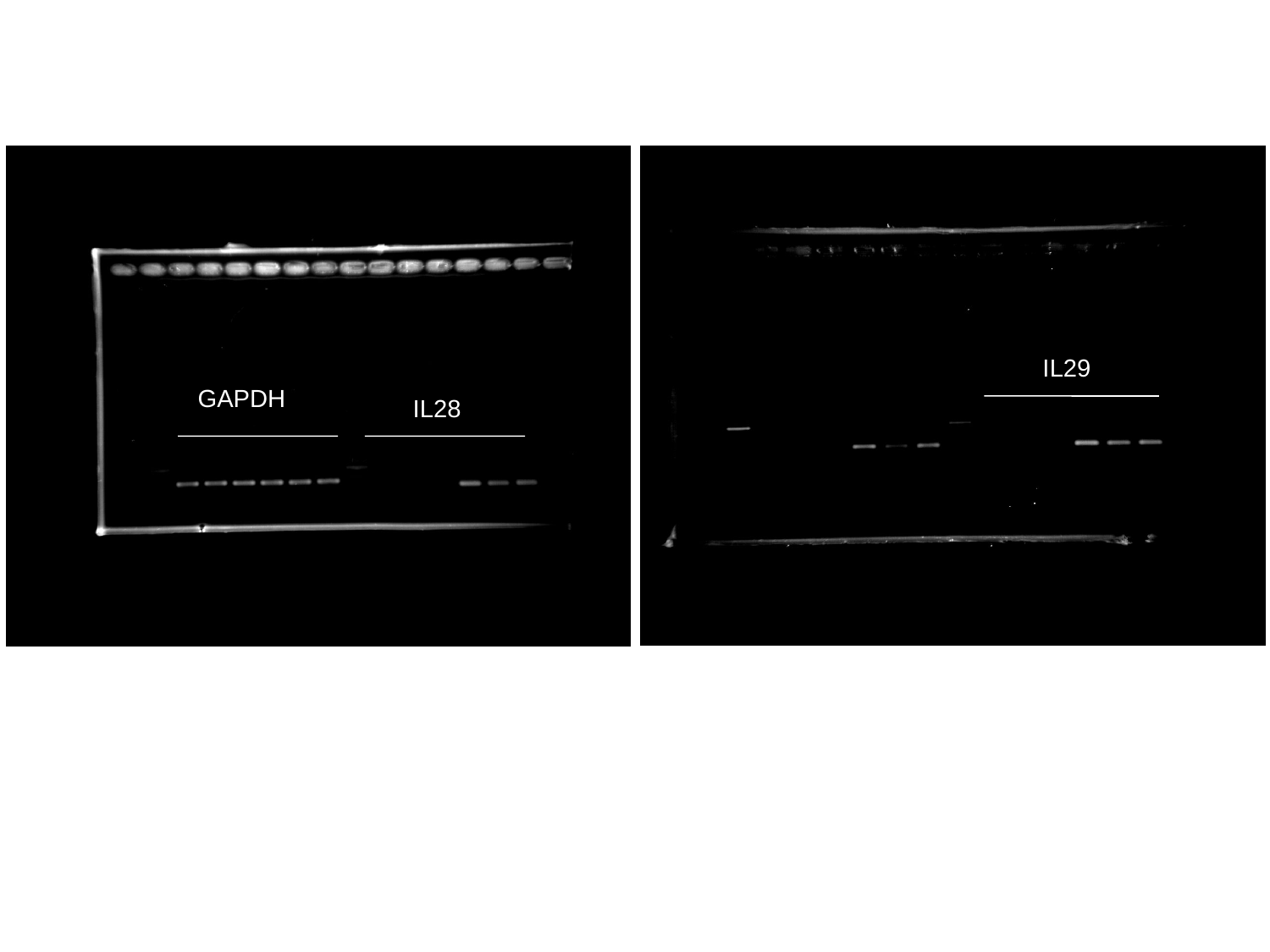

GAPDH
IL28
IL29

Supplement: S3 Fig — A549 cell lines stably expressing STAT1-WT, STAT1-2C or empty vector (EV) were infected with or without WSN virus for 15 h. mRNA levels of IL-28A/B, GAPDH and IL-29 were measured by RT-PCR and examined by agarose gel electrophoresis. (PPT) [file ppat.1005402.s003.ppt]

## Slide 1
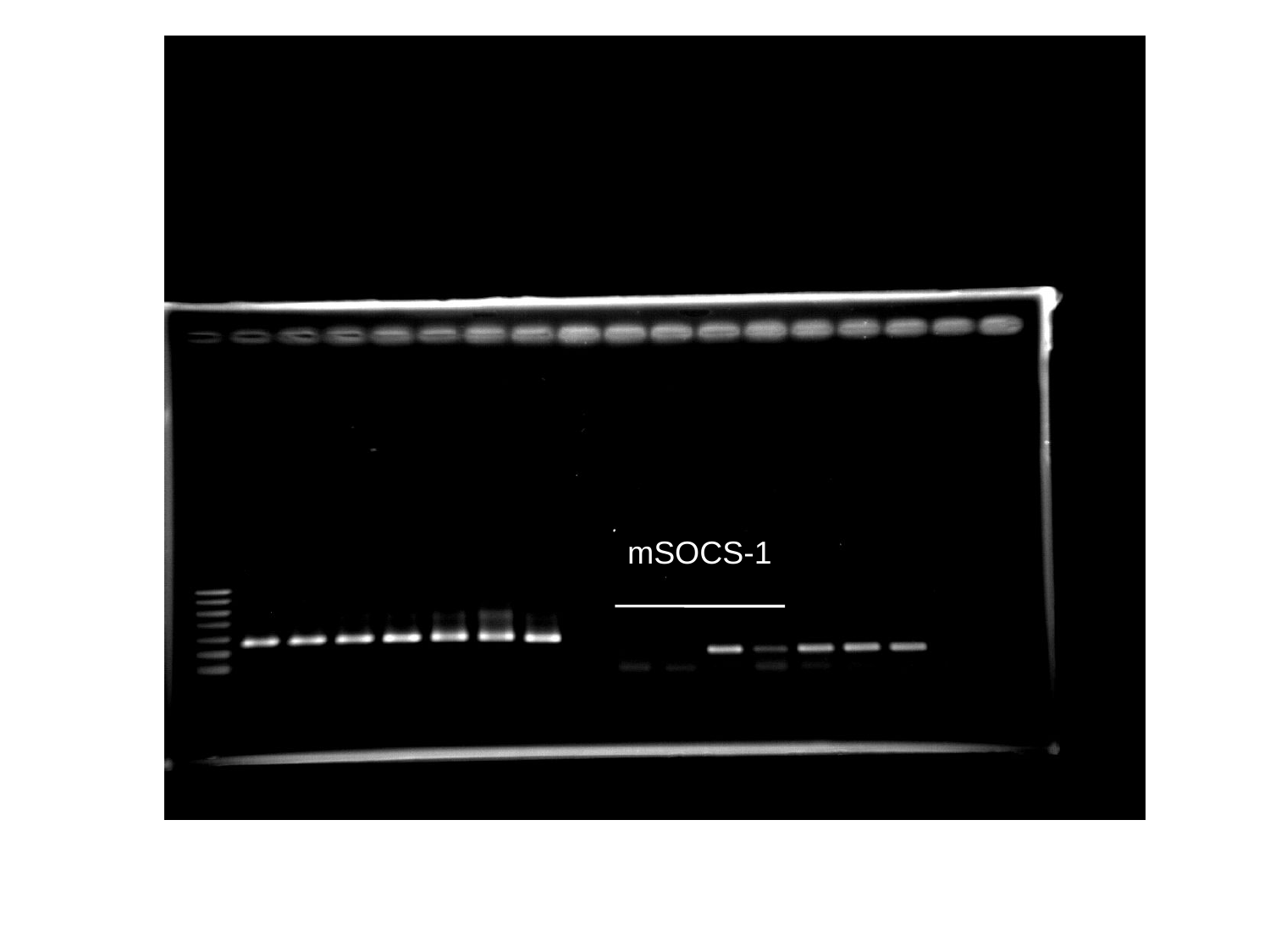

mSOCS-1

## Slide 2
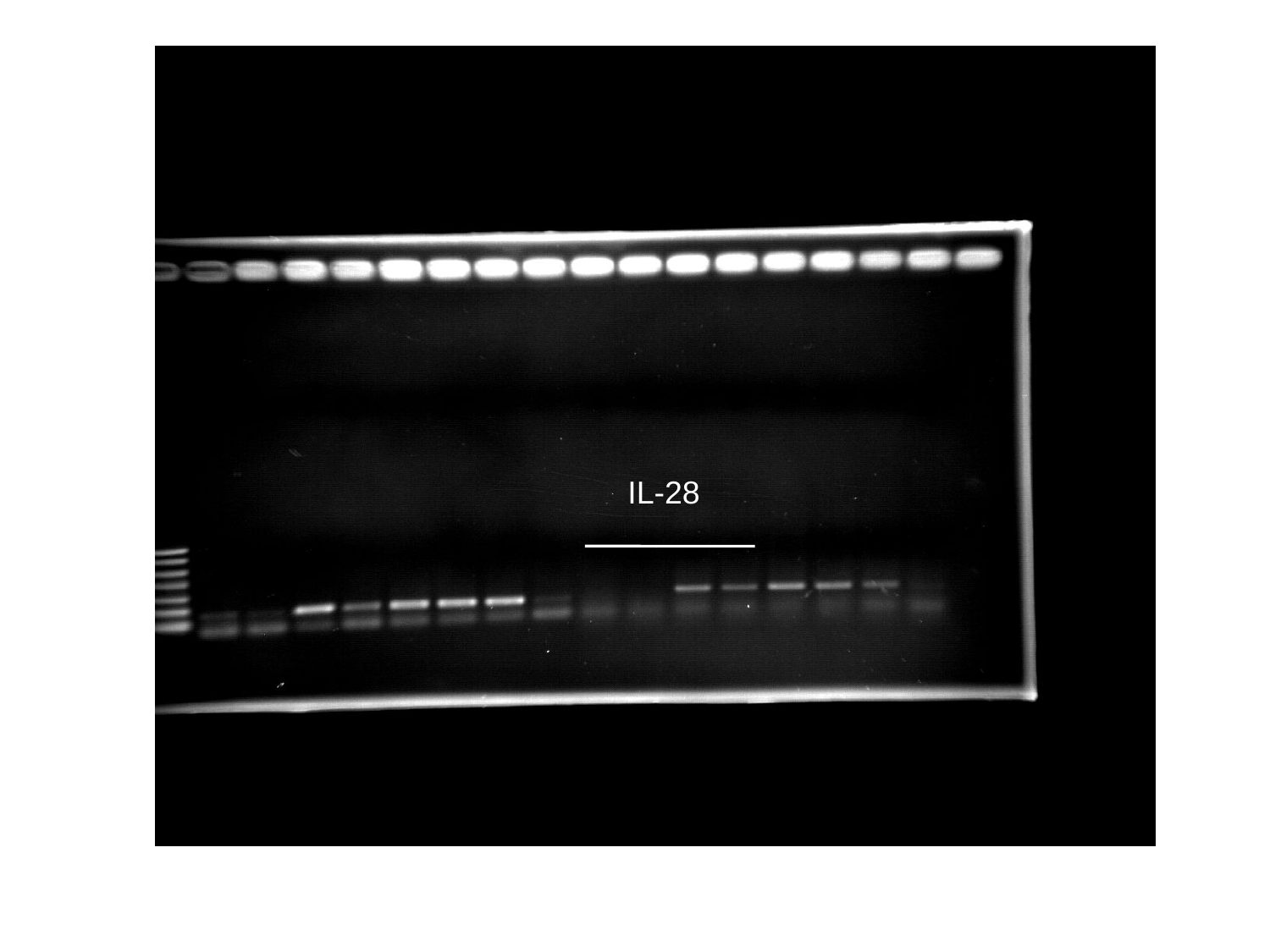

IL-28

## Slide 3
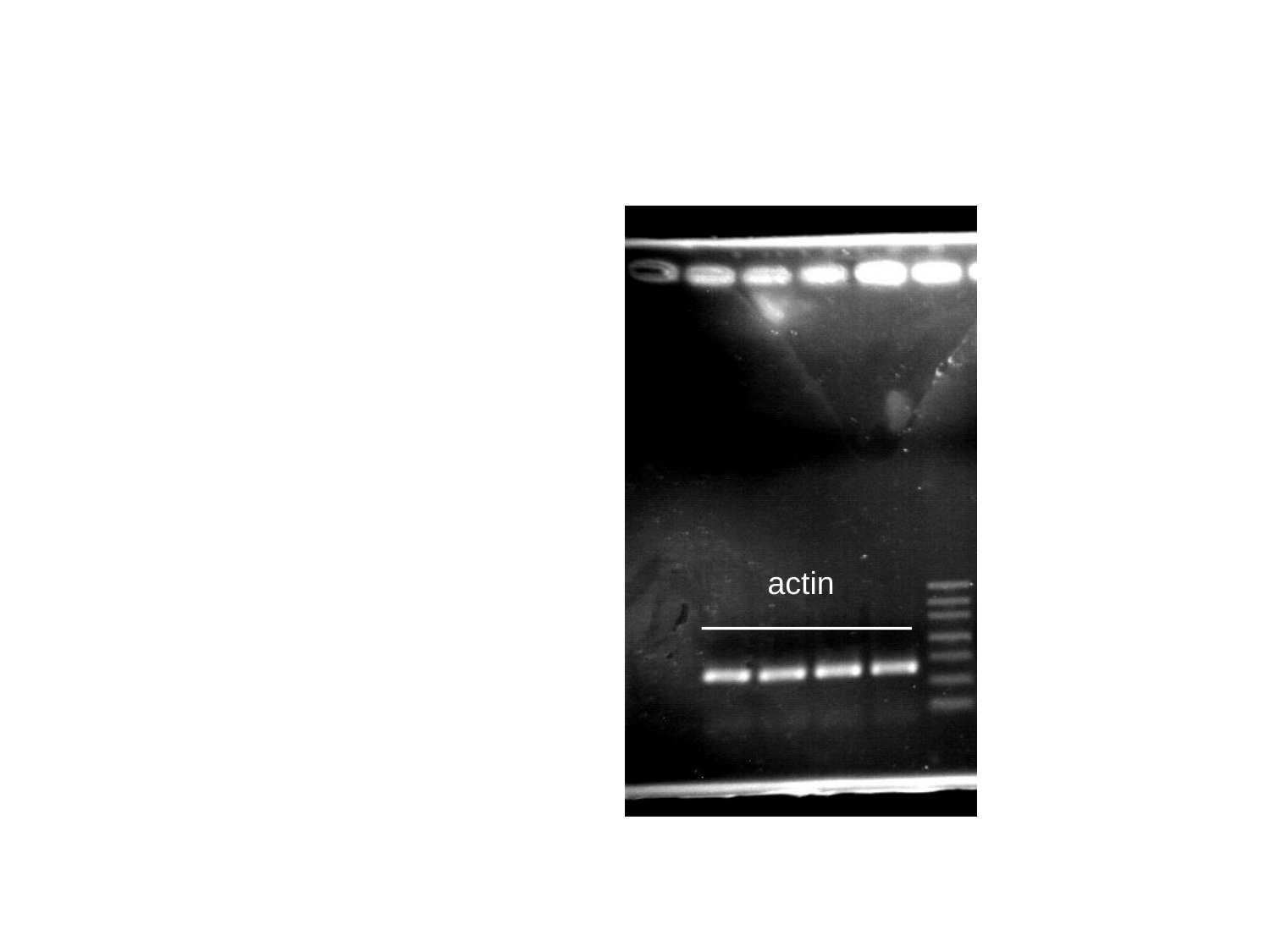

actin

Supplement: S4 Fig — WT and TG mice were infected with or without WSN virus intranasally (1×10^5^ PFU). On Day 3 p.i., lungs were lysed and expression of IL-28A/B, mSOCS1 and β-actin was examined by RT-PCR followed by agarose gel electrophoresis. (PPT) [file ppat.1005402.s004.ppt]
